# The costs of transgenerational immune priming for homologous and heterologous infections with different serotypes of dengue virus in *Aedes aegypti* mosquitoes

**DOI:** 10.3389/fimmu.2023.1286831

**Published:** 2023-12-18

**Authors:** Jorge Cime-Castillo, Valeria Vargas, Juan Manuel Hernández-Tablas, Edgar Quezada-Ruiz, Grecia Díaz, Humberto Lanz-Mendoza

**Affiliations:** ^1^ Infection and Immunity Direction/Vector Borne Disease Department, Centro de Investigaciones Sobre Enfermedades Infecciosas-Instituto Nacional de Salud Pública (INSP), Cuernavaca, Mexico; ^2^ Biomedical Research Institute, Universidad Nacional Autonoma de México, Ciudad de México, Mexico

**Keywords:** transgenerational immune priming, *Aedes aegypti*, dengue virus, vector-borne diseases, trade-off

## Abstract

The immune system is a network of molecules, signaling pathways, transcription, and effector modulation that controls, mitigates, or eradicates agents that may affect the integrity of the host. In mosquitoes, the innate immune system is highly efficient at combating foreign organisms but has the capacity to tolerate vector-borne diseases. These implications lead to replication, dissemination, and ultimately the transmission of pathogenic organisms when feeding on a host. In recent years, it has been discovered that the innate immune response of mosquitoes can trigger an enhanced immunity response to the stimulus of a previously encountered pathogen. This phenomenon, called immune priming, is characterized by a molecular response that prevents the replication of viruses, parasites, or bacteria in the body. It has been documented that immune priming can be stimulated through homologous organisms or molecules, although it has also been documented that closely related pathogens can generate an enhanced immune response to a second stimulus with a related organism. However, the cost involved in this immune response has not been characterized through the transmission of the immunological experience from parents to offspring by transgenerational immune priming (TGIP) in mosquitoes. Here, we address the impact on the rates of oviposition, hatching, development, and immune response in *Aedes aegypti* mosquitoes, the mothers of which were stimulated with dengue virus serotypes 2 and/or 4, having found a cost of TGIP on the development time of the progeny of mothers with heterologous infections, with respect to mothers with homologous infections. Our results showed a significant effect on the sex ratio, with females being more abundant than males. We found a decrease in transcripts of the siRNA pathway in daughters of mothers who had been exposed to an immune challenge with DV. Our research demonstrates that there are costs and benefits associated with TGIP in *Aedes aegypti* mosquitoes exposed to DV. Specifically, priming results in a lower viral load in the offspring of mothers who have previously been infected with the virus. Although some results from tests of two dengue virus serotypes show similarities, such as the percentage of pupae emergence, there are differences in the percentage of adult emergence, indicating differences in TGIP costs even within the same virus with different serotypes. This finding has crucial implications in the context of dengue virus transmission in endemic areas where multiple serotypes circulate simultaneously.

## Introduction

Pathogens can cause damage to their hosts through infection that occurs when they invade an organism. The host, on the other hand, reacts to the invasion through a series of immune response molecules, which depends mostly on the severity of the infection and previous encounters with the pathogen. There are two main types of immune response: innate and adaptive (1). The adaptive response in vertebrates is based on the clonal selection of cells with specific receptors (such as lymphocytes). This response has evolved from mandibulated organisms, showing vast differences in the immune response of organisms below this phylogenetic organization (2, 3). However, it has been demonstrated that several invertebrate organisms show an adaptive immune response (which does not involve lymphocyte clonal selection) to a pathogen that had previously been encountered during its life stage (4, 5). This phenomenon, known as immune priming, involves a more efficient response after a first encounter. Additionally, this response can occur with a broader group of pathogens (6–8).

It should be noted that in a second encounter with pathogens, most published papers have shown better immune protection than the first time (9). Interestingly, in some studies, immunological costs have been recorded through the development, survival, or fecundity of host organisms (reviewed in 10).

The immune priming larvae stage can enhance adult immunity (within generational protection (11–14)), and even transgenerational immune priming (TGIP), which refers to the transfer of parental immunological experience to its progeny and has been described in several models (15–23), in which the enhanced immune response has been observed over one or several generations. However, the impact of transgenerational responses to immune priming has not been fully explored, despite the potential consequences for offspring reproductive success. Previous studies have demonstrated that such trade-offs can include reduced offspring survival, longevity, development time, fecundity, and sperm viability (20, 24–27). In *Anopheles albimanus* mosquitoes infected with *Plasmodium berghei*, improved immune responses also result in decreased hatching rates in primed mosquitoes compared with untreated individuals, and there is a trade-off between parasite elimination and egg production in primed females (28).

Mosquito vectors of pathogens of medical importance are a widely studied group due to the transmission of viruses and parasites with high incidences of morbidity and mortality throughout the world. For decades, the eradication or control of mosquito populations has been a key strategy for decreasing the likelihood of contracting infectious agents (29, 30). Therefore, the immune response of mosquitoes has been a topic of interest over the years as it is important to fully understand their biology and ecology in order to propose strategies for transmission control (31). *Aedes aegypti* and *Anopheles gambiae* are the best-studied mosquitoes in which various immune response pathways have been detected in the face of challenges defining specific response routes for gram-positive and gram-negative bacteria, viruses, parasites, and fungal stimuli. Immune generational priming has been described in both mosquitoes, and an improved immune response to a second encounter with the same pathogen has been documented in *Aedes aegypti* primed with dengue virus (32, 33) as well as in *Anopheles* primed with *Plasmodium berghei* (34, 35).

However, few studies have examined the protection from viruses in arthropods (36) or the induction of TGIP by viral infection (23, 37). Injecting β-1,3-1,6-glucan into shrimp (*Penaus monodon*) resulted in an increase in the relative percentage survival of larvae compared with untreated groups, indicating that maternally transmitted disease resistance induced by glucan protects larvae from viral infection associated with white spot syndrome (36). In *Plodia interpunctella*, exposure to a low dose of *Plodia interpunctella* granulovirus, a DsDNA virus, reduces the subsequent susceptibility to lethal viral infection in the offspring of exposed parents (37). In *Drosophila melanogaster*, it was observed that the offspring of mothers primed with Sindbis virus had less viral replication. Protection was absent in unrelated viruses, such as cricket paralysis virus (CrPV), and TGIP is widely distributed among single-stranded positive-sense RNA viruses. In the same study, TGIP was described in *Aedes aegypti* mosquitoes against chikungunya virus (23). These studies have demonstrated the role of TGIP in reducing viral load. However, a homologous and/or heterologous challenge with a virus with different serotypes, such as the dengue virus, has not yet been tested. Likewise, the costs before homologous and heterologous immunological priming with dengue virus are unknown. In this investigation, we assessed the impact of TGIP, utilizing both homologous and heterologous challenges with two dengue virus serotypes, on the fitness of its mosquito vector. This analysis will offer insights into the biology and immune response of the mosquito in the event of exposure to multiple serotypes, which is of significance for the transmission dynamics of dengue virus in areas where multiple serotypes co-circulate. Research has reported data on the adaptation and survival of *Aedes aegypti* mosquitoes in response to viral infections (38). However, the costs associated with homologous and heterologous immunological priming with dengue virus remain unknown.

## Methods

### Mosquito rearing

The *Ae. aegypti* Rockefeller strain of mosquito was maintained under controlled insectary conditions at the Instituto Nacional de Salud Pública (INSP), Mexico. Standard breeding conditions of the mosquitoes were a temperature of 27–29°C, relative humidity of 60–80%, and photoperiod of 12:12 light/dark cycle. Larvae were fed a standard mix diet of yeast extract, lactalbumin, and mashed mouse food pellets (LabDiet, 5008) in a 1:1:1 (m/m) ratio and were reared in plastic trays in 2 L of dechlorinated tap water at a density of approximately 200 larvae until pupation. Pupae were transferred into cages for adult emergence. Adult mosquitoes were maintained with a 10% sucrose solution. At 5 days post-emergence, the sucrose solution was discarded up until 12 h before infection with rabbit blood + dengue virus (DV) or rabbit blood-feeding (RBF) alone.

### DV propagation and titration

DV New Guinea C strain serotype 2 (DV2, initially donated by Dr. Duane Gubler, CDC Fort Collins, CO, USA and kept in our virus bank at the Infectious Diseases Research Center) and DENV serotype 4 (DV4, obtained from a febrile patient and kindly donated by Dr. Rosa Ma. Del Angel, CINVESTAV, IPN) were propagated in C6/36 cells (*Ae. albopictus*, larvae cells), which were grown at 28°C in Schneider´s insect medium (Sigma-Aldrich). Confluent monolayers were infected for 2 h at a multiplicity of infection (MOI) of 1 and incubated for 5–7 days at 28°C in a 5% CO2 atmosphere until cytopathic effects were observed before titrating in a lytic plaque assay using LLC-MK2 cells (monkey, rhesus *Macaca mulatta*, epithelial kidney). The virus titer was expressed as plaque-forming units (pfu) per milliliter. The selection of serotypes is based on the fact that DV-4 has been shown to be the most genetically distant serotype from the other three serotypes (DV-1-3) [ (39, 40). **Figure S1**]. Both serotypes have been previously used for immune priming in our laboratory (13, 32).

### Maternal immune priming treatment

The maternal female and offspring generations were reared under the same conditions as described above. For maternal immune priming (F0) treatments, adult mosquitoes were used 5 days after emergence.

**Figure 1:**
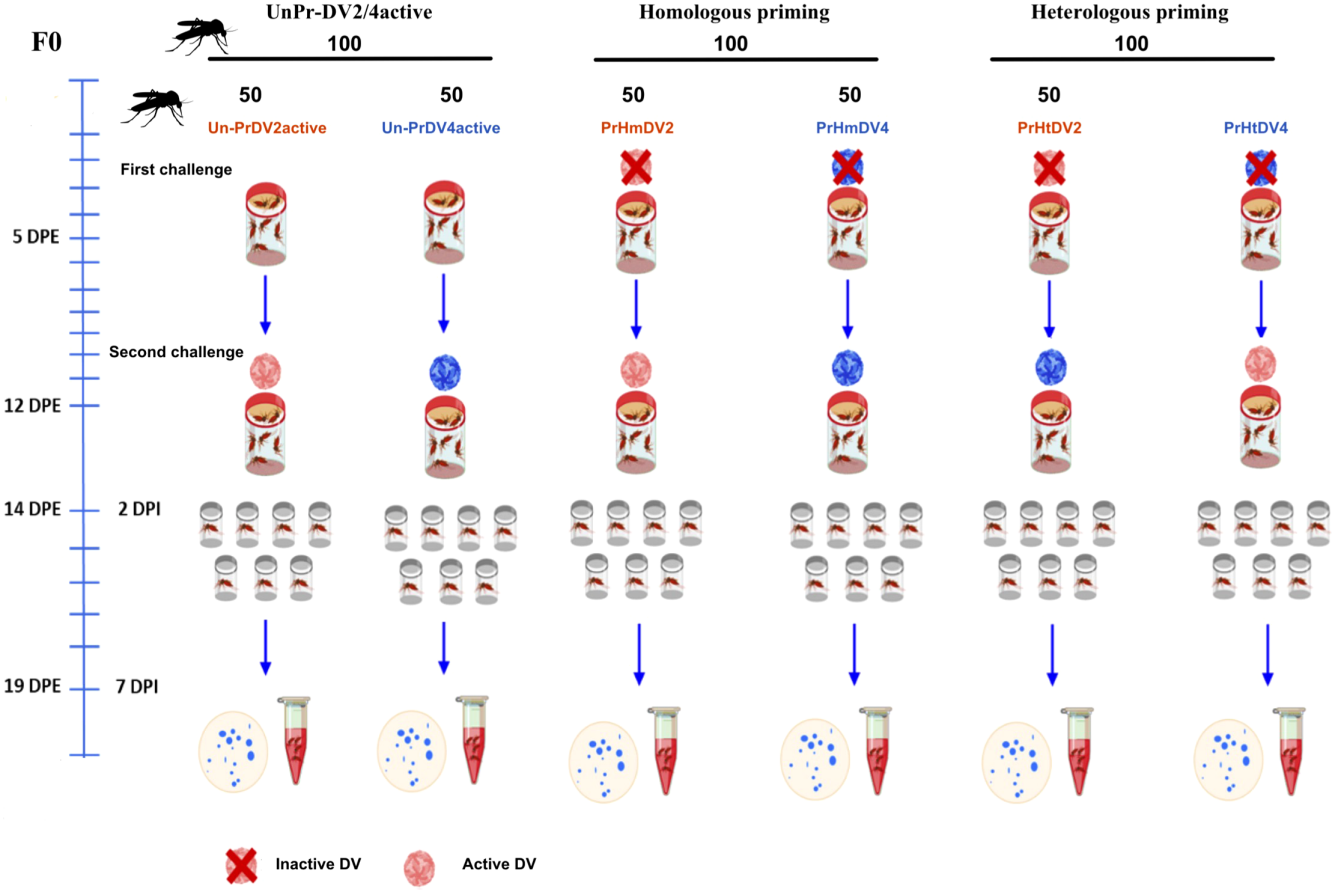
Experimental timeline of immune primed-mother mosquitoes of *Aedes aegypti* (F0) challenged with heterologous and homologous infections with dengue virus serotypes 2 (DV2, represent in red) and 4 (DV4, represent in blue), as well as unprimed mothers with dengue virus (UnPr). At five days post-emergence (DPE), the first immune challenge with inactive dengue virus (inactive DV) was performed for homologous and heterologous infections. Mothers not immunized with the dengue virus were fed with rabbit blood. The second immune challenge was performed at seven days post-infection (DPI). The mosquitoes were infected with active dengue virus (active DV), both for priming homologous, heterologous, and UnPr mosquitoes. At 14 DPI, individual mosquito excreta were collected to confirm DV infection by real-time PCR. The blue spots represent the individual excreta.

The immune challenge consisted of two stages: i) the induction of maternal immune priming using inactive dengue virus serotypes 2 (DV2) and 4 (DV4)) with UV light; and ii) the second immune challenge was performed 7 days post-immune priming using the same serotype infections with active dengue virus of the immune priming (homologous infections; PrHmDV2 or PrHmDV4, respectively) or with a different serotype of dengue virus of the immune priming (heterologous infections; PrHtDV2 or PrHtDV4, respectively). For oral infection, rabbit blood (RBF) and previously titrated DV2 or DV4 were mixed at a 1:1 (v/v) ratio. This mixture was placed in glass chambers covered with parafilm M, recirculating water at 37°C (**Figure 1**).

**Figure 2:**
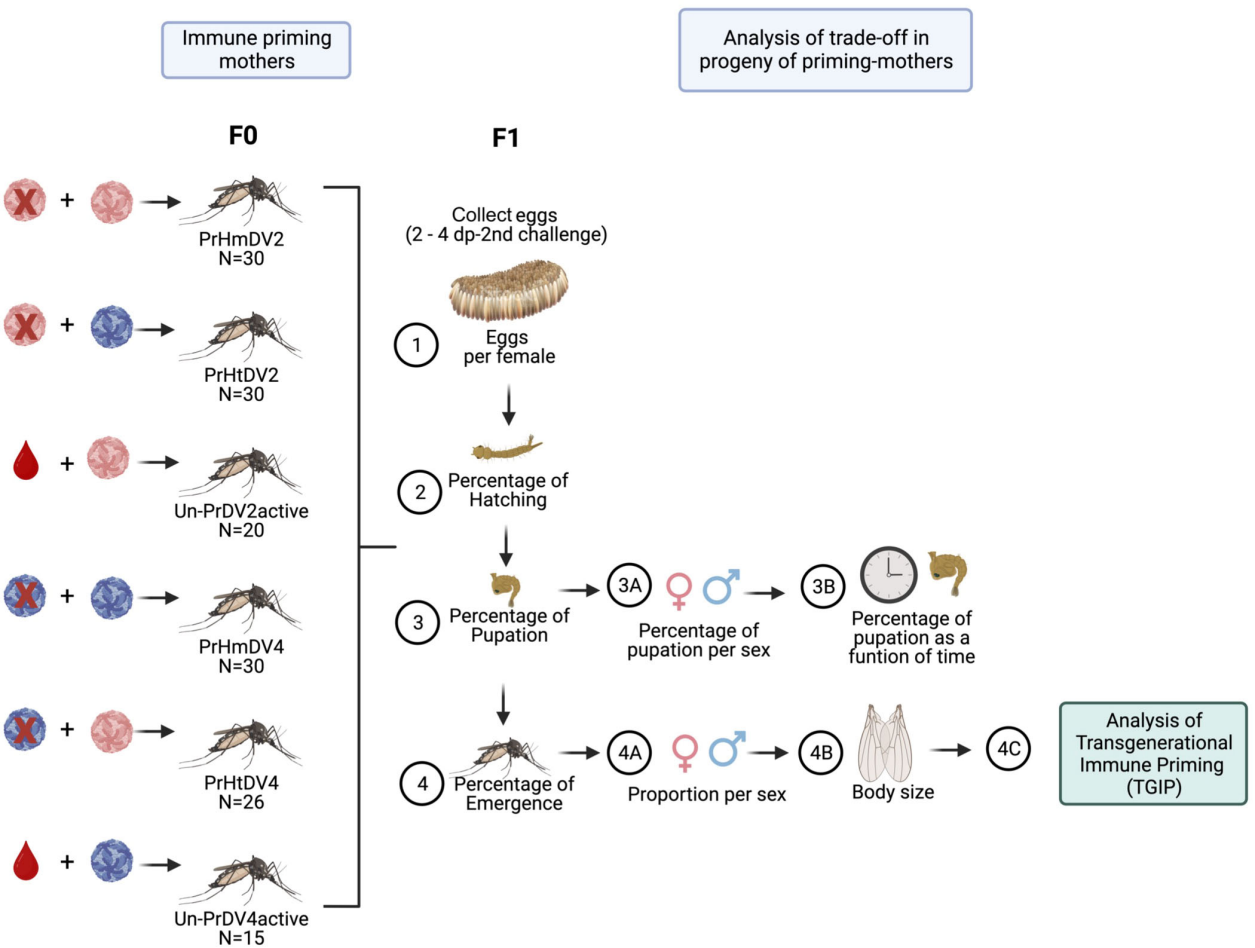
Experimental design of the trade-off analysis of the progeny (F1) of priming mothers (F0) with homologous (PrHm), heterologous infection (PrHt) infection with dengue virus serotype 2 (represented in red), 4 (represented in blue) and unprimed mosquitoes (UnPr), which evaluated different biological parameters: 1) Oviposition (number of eggs deposited by individual mothers); 2) Percentage of Hatching (percentage of the number of eggs hatched); 3) Percentage of pupation (percentage of the number of larvae that pupated); 3A) Percentage of pupation by sex (percentage of number of larvae that pupated in both sexes); 3B) Percentage of pupation as a function of time (Percentage of the number of larvae that pupated over time); 4) Percentage of emergence (Percentage of the number of pupae that emerge to adult); 4A) Sex ratio (number of males and females that emerge to adult); 4B) Body size (measurement of the pair of wings of adult mosquitoes) and 4C) Transgenerational immune priming (TGIP) analysis (See **Figure 3**).

For the first challenge of F0 mothers (immune priming), 200 female mosquitoes were fed orally with rabbit blood + light-UV-inactivated DV2 or DV4 for 3 h at 4°C (supernatants from infected C6/36 cells with a titer of 1x10^2^ RNA copies/ml. **Figure S2**). The other 100 female mosquitoes were fed orally with RBF for unprimed groups. After feeding, fully engorged females were separated and placed in new cages until the next challenge. Six groups of 50 mosquitoes per individual treatment were formed: two groups of homologous priming (PrHmDV2 and PrHmDV4); two groups of heterologous priming (PrHtDV2 and PrHtDV4); and two groups of unprimed maternal females (UnPrDV2 and UnPrDV4). Seven days post-feeding, all groups were challenged with DV2 and/or DV4 (supernatants from infected C6/36 cells with a titer of 1x10^6^ RNA copies/ml) (**Figure 1**). Only fully fed females were considered after the first and second feeding, leaving the groups indicated in **Figure 2**. Females not fully engorged were not included in the analysis. No mortality was observed in F0 and F1 females.

Two days after the second challenge, oviposition containers were placed for each female. A filter paper disk was placed below each plastic container, and cotton soaked in a honey-colored solution was placed on top of the container. The honey-colored solution consisted of 5 g of bee honey diluted in 100 μl of blue food dye (Deiman, Mexico) and 10 ml of sterile tap water. This solution allowed the visualization of colored excreta spots on the filter paper (**Figure 1**). The virus in mosquito excreta was detected at 7 and 14 days post-infection, as has been evaluated previously (13). A follow-up study was conducted to determine viral load levels up to 5 days post-infection.

### Offspring rearing and treatment

**Figure 3:**
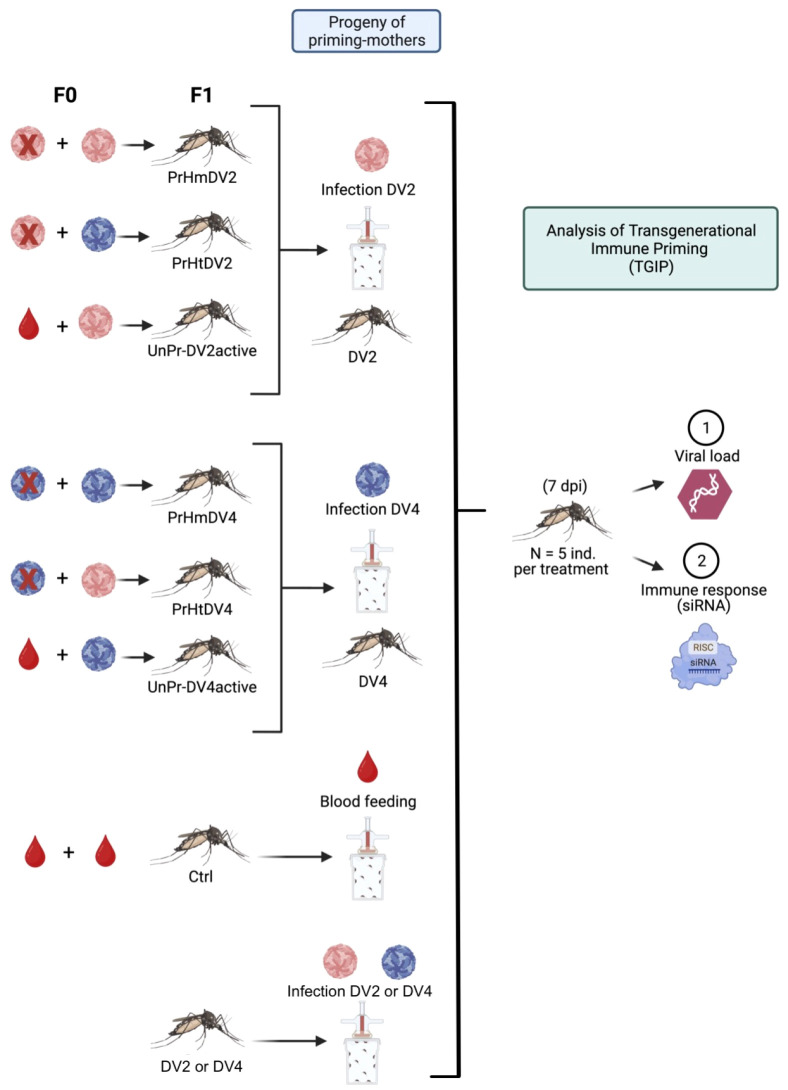
Experimental design of the transgenerational immune priming (TGIP) analysis of the progeny (F1) of priming mothers (F0) with homologous (PrHm) and heterologous (PrHt) infections with dengue virus serotypes 2 (DV2, represented in red) and 4 (represented in blue) and unprimed (UnPr) mosquitoes but infected with a second challenge with active dengue virus (UnPr-DV2active or UnPr-DV4active respectively). As DV infection control groups, progeny (of mothers without infection with DV) were infected with DV2 or DV4, respectively. In addition, another control group fed with rabbit blood without infection (Ctrl) was added. At seven days post-infection (dpi), five individuals per treatment were collected to evaluate the viral load and antiviral immune response (siRNA).

The hatching of eggs was carried out by placing tap water into the individual oviposition containers for 24 h in insectary conditions. The following day, the newly hatched larvae (L-1) were placed in 300 ml of tap water in small plastic cups (diameter of 11 cm, and a height of 7.5 cm) coming from each mother female. Larvae (not more than 125 per container) were maintained in these cups until the pupal stage. Then, the pupae were placed in a smaller plastic cup (diameter 4 cm; height 4 cm) in a large plastic container (diameter of 11 cm, and a height of 14.5 cm) coming from each mother female until mosquitoes developed to adulthood (**Figure 2**). F1 adults 5 days post-emergence were used for the priming treatment. Mosquitoes from primed mothers were infected with the same dengue virus serotype that the mothers had been infected with previously (F0). For example, offspring from primed mothers with homologous infections with serotype 2 or 4 (PrHmDV2 or PrHmDV4, respectively) were challenged with the same serotype that their mothers had been primed with previously (PrHmDV2/DV2 or PrHmDV4/DV4, respectively) (**Figure 3**). Likewise, F1 adults from primed mothers with heterologous infections with serotype 2 or 4 (PrHtDV2 or PrHtDV4, respectively) were challenged with the same serotype that their mothers had been primed with previously (PrHtDV2/DV2 or PrHtDV4/DV4, respectively) (**Figure 3**). Furthermore, adult F1 from unprimed mothers who were challenged with serotypes 2 or 4 active (UnPr-DV2active or UnPr-DV4active) were challenged again with the same serotype that their F0 mothers had previously been infected with (UnPr-DV2/DV2 or UnPr-DV4/DV4). For infection control, F1 adults from untreated mothers (unprimed-no challenge) were challenged with DV2 or DV4. For not-infection control, F1 adults from untreated mothers were fed only with RBF (Ctrl; **Figure 3**).

### The oviposition and fecundity parameters of primed mothers

To elucidate the benefits and costs of the maternal priming treatment, the following parameters were recorded: the number of eggs that were laid per female and the hatching percentage of the eggs. These parameters allow the evaluation of not only the oviposition but also the fertility and viability of the eggs (**Figure 2**).

To estimate the number of eggs, a filter paper was placed in each oviposition container for the females to lay their eggs. The filter paper was left for 3 days in the female cages to evaluate egg laying and was then removed and left to dehydrate together with the eggs for 2 days until later use. The quantification of the eggs was carried out using image processing and analysis in Java (ImageJ bundled with Java 1.8.0_172). To quantify the percentage of egg hatching, 20 ml of tap water was added to the oviposition containers for 24 h. The number of newly hatched larvae (L-1) per treatment was estimated using the following formula: the total number of larvae for each female per treatment divided by the total number of eggs of each female per 100%. These parameters were compared between unprimed mothers (UnPr-DV2active or UnPr-DV4active), homologous mothers (PrHmDV2 or PrHtDV4), heterologous primed mothers (PrHtDV2 or PrHtDV4), and the control group (Ctrl).

### Offspring developmental time, percentage of pupation, the emergence of adults, and wing size parameters

A total of N = 6,083 larvae were obtained, which were divided into UnPr-DV2active (N = 611), UnPr-DV4active (N = 661), PrHmDV2 (N = 1122), PrHmDV4 (N = 1157), PrHtDV2 (N = 965), PrHtDV4 (N = 677), and Ctrl (N = 890) groups. All the offspring were monitored daily for pupation to quantify development time, the percentage of pupation, the emergence of adults, and body size parameters (**Figure 2**).

To estimate the pupae development time, the number of pupae were quantified and separated by sex every day; the monitoring was performed until the last larvae were developed in each treatment. The larvae that died in the development process were discarded from the study. To separate the pupae by sex, the body sizes of five female and male pupae were measured, as described by Bellini et al., 2018 (41) (**Figure S3**). After separating the pupae by sex, the mosquitoes were placed in plastic containers for the verification of the previous sex determination in the pupae already in the adult stage by the morphological characters determined for each one. To evaluate the percentage of pupation, the following formula was used: the total number of pupae (female + male) divided by the total number of larvae in each treatment, multiplied by 100. To estimate the percentage of emergence, the number of adults that emerged was quantified until the last adult mosquito emerged. The pupae that did not emerge as adult mosquitoes were discarded from the study. The percentage of emergent female mosquitoes was estimated using the following formula: the total number of adult females divided by the total number of pupae per treatment, multiplied by 100. Likewise, we used the same formula to estimate the percentage of emergent male mosquitoes. Body size was estimated by measuring the size of both wings of 20 individual females per treatment. The wings were detached and mounted on a paper sheet; a photograph was taken of each wing at a magnification of 10× using a camera (Canon EOS 50D). A centimeter ruler was photographed to standardize size measurements through ImageJ software. These parameters were compared between all treatments and the control group.

### Offspring susceptibility and antiviral immune response against dengue virus

Following the emergence of offspring adult mosquitoes (F1) after 5 days, the challenge was applied either homologously or heterologously, as described above. At 7 days post-challenge, individual female mosquitoes per treatment were collected to quantify the susceptibility of the dengue virus and analyze the relative expression of the antiviral immune response against the virus (**Figure 3**). To assess the replicability of the phenotypic patterns, we conducted an additional experiment involving the measurement of the viral load and gene expression levels of representative RNAi pathway genes at day 5 post-infection under the same priming/challenge conditions. This experiment was conducted with 15 mosquitoes per group, with only fully fed females being taken into consideration for analysis.

To assess the susceptibility of offspring to dengue virus, RNA was extracted to estimate the viral load of individual mosquitoes. Total RNA from individual mosquitoes was extracted using a Qiamp Viral RNA mini kit (Qiagen), according to the manufacturer’s instructions. In the final step, the total RNA from each sample was eluted in 60 μl of RNase-free water, and the RNA concentration was measured using a NanoPhotometer NP80 (Implent). We normalized 500 ng/μl of the total RNA treated with 0.5 μl of DNase, 0.8 μl buffer DNase (Thermo Scientific), and H_2_0 DEPC to a final volume of 8 μl for cDNA synthesis. To synthesize cDNA, we used 1 μl of RevertAid Reverse Transcriptase (20 U/μl; Thermo Scientific), 1 μl of RiboLock RNase Inhibitor (40 U/μl; Thermo Scientific), 1 μl of random hexamer primer (100 μM; Thermo Scientific), and 1 μl of dNTPs (10 μM; Thermo Scientific). We used 1 μl of cDNA for real-time quantitative PCR reactions using Maxima SYBR Green/ROX qPCR Master Mix (Thermo Scientific) on a Rotor-Gene 5Q (Qiagen). For absolute quantification, a standard curve was generated using a 10-fold serial dilution of a synthetic gene (gBlock) of known concentration to DV (2.7 × 10^10^ Dengue genome copies per microliter; Integrated DNA Technologies). The total viral load per mosquito was extrapolated from CT values using least-squares fitting (Rotor-Gene Q series, version 2.3.4; Software). The gBlock was designed in a 5`untranslated region and in a portion of the capsid protein to amplify 350 base pairs (Integrated DNA Technologies). In addition, primers were designed to amplify 200 bp of the dengue virus envelope protein (**Table 1**). Using the absolute quantification curve, generated through the use of the synthetic dengue virus gene, we determined that the minimum detectable viral concentration lies at a CT value of less than 33.5.

**Table 1:** List of qPCR primers used in the experiments.

Primers for qRT-PCR	Sequence (5′–3′)
**DV_all (Fw)**	**CAA TAT GCT GAA ACG CGA GAG AA**
**DV_all (Rv)**	**CCC CAT CTA TTC AGA ATC CCT GC**
**AGO2 (Fw)**	**CAG TGC GTT CAG GCC AAA AA**
**AGO2 (Rv)**	**TCC ACC CAG TTT GAC GTT GA**
**DCR2 (Fw)**	**CGA AGA GGT CAT TGG TGG CT**
**DCR2 (Rv)**	**CAC GGC AGA GGT ATA TCG CC**
**R2D2 (Fw)**	**CAC TTT TTG GCG GTC CTG TC**
**R2D2 (Fw)**	**TTC GGG GCA TCT CGA AGT TC**
**S7 (Fw)**	**GGG ACA AAT CGG CCA GGC TAT C**
**S7 (Rv)**	**TCG TGG ACG CTT CTG CTT GTT G**

Gene expression was assayed by RT-qPCR using a Rotor-Gene 5Q (Qiagen). The qPCR master mix contained 0.4 μM of each primer, 5 μl of 1× Maxima SYBR Green/ROX (Thermo Scientific), 1 μl of cDNA template, and 3.2 μl of RNase-free water for a final 10 μl volume. Specific primers for the siRNAs, miRNA pathways, and peptide antimicrobial were designed as markers for the antiviral immune response after the second challenge. The primers used for the amplification of Argonaute-2, Dicer-2, R2D2, ribosomal protein S7, and DV are listed in **Table 1**. The relative quantification of mRNA levels was carried out using the 2^−ΔΔCT^ method, and primer efficiencies were calculated by measuring how the standard *ΔCT* varied with template serial dilutions (PCR efficiency was approximately 95–99% for each primer). For all assays, the ribosomal protein *S7* gene was used as the reference. Levels of AGO2, DCR2, and R2D2 were normalized with regard to the *S7* transcript of the same sample. Melting curve analyses confirmed that only cDNA, and not genomic DNA, was amplified. Therefore, we standardized these differences in copy numbers in ratios for all primers. Three independent assays were conducted, each analyzed in duplicate.

### Statistical analysis

To analyze the normal distribution of i) oviposition, ii) hatching percentage, iii) percentage of pupation, iv) percentage of emergence, and v) relative expression of mRNA involved in the antiviral immune response per treatment, we carried out Anderson–Darling, D’Agostino–Pearson omnibus Shapiro–Wilk, and Kolmogorov–Smirnov tests. (**Supplementary Tables 1** and **2**. Relative expression of mRNA and biological parameters). To elucidate the maternal immune priming cost, the effects of offspring fitness, and mRNA relative expression, we performed a one-way ANOVA for oviposition, hatching percentage, percentage of pupation, and percentage of emergence. A chi-squared test was used for sex ratio, and a two-way ANOVA was used for the relative expression of mRNA involved in the antiviral immune response per treatment. All ANOVA analyses indicated a significant difference between treatments, and thus, we carried out pair comparisons using a correct Tukey’s multiple comparison test. We include three tables (biological parameters, sex ratio, and the relative expression of mRNA) in the **supplementary material** (**Supplementary Tables 3**-**6**) showing the value of significant differences between treatments and pairwise comparisons (a chi-square test and Tukey’s multiple comparisons test). These statistical analyses were performed in GraphPad Prism version 6 Oc. (CA, USA). The number of pupae per day was adjusted to a Weibull NHPP model of different systems and evaluated using a chi-square. The survival curve was compared with a normal distribution and homogeneity test as a function of weighting (log-rank). The survival proportion was calculated through a maximum likelihood estimate of the survival [Peto, 1973 (42)] and the number of risks through a Wilcoxon test. To identify patterns and structure in the data, and reveal the relative importance of individual variables, we performed a principal components analysis (PCA). The viral load, gene differential expression, emergence, oviposition, and pupation from all experimental conditions (Ctrl; UnPr-DV2 and DV4; PrHm DV2 and DV4 and PrHt DV2 and DV4) were included in PCA analysis. The survival proportion and PCA were conducted using the statistical discovery software JMP 16.

## Results

To determine whether immune priming affects the progeny of mosquitoes, we evaluated the first generation of mosquitoes from mothers challenged with dengue virus serotype 2 or dengue virus serotype 4. The challenge was applied in a homologous or heterologous manner with dengue virus inactivated by ultraviolet light as the first immunological challenge (immune priming) and active virus as the second challenge. Each of the challenged females was provided in a particular container of an oviposition tray, and their offspring were evaluated for the following parameters: number of eggs per treatment, hatching rate, percentage of pupation, number of adults that emerged, percentage of sexes, and estimation of body size (**Figure 2**). Once the adults (F1) were obtained, an immune challenge was applied with DV2 or DV4 (**Figure 3**), and the viral load and molecules of the immune response were evaluated.

### Effect of immune priming on oviposition and hatching

**Figure 4:**
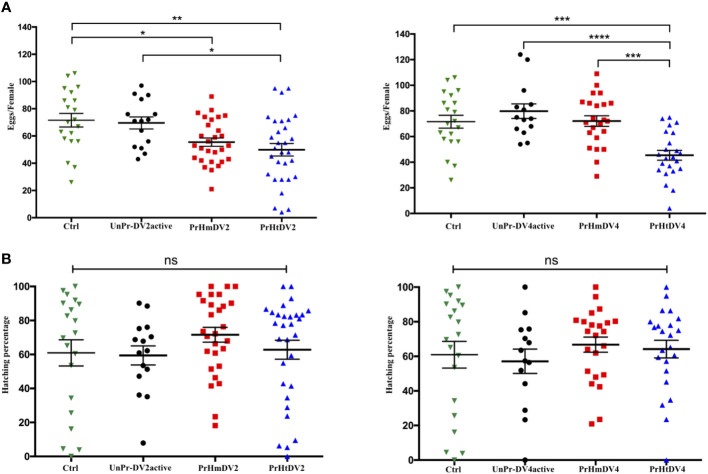
Analysis of **(A)** Oviposition (number of eggs per female) and **(B)** Percentage of hatching of eggs from mothers of *Ae. aegypti* (F0), previously immunized with dengue virus serotypes 2 (DV2) and 4 (DV4). PrHm (priming-mothers with DVinactive and challenged with DVactive with the same serotype); PrHt (priming-mothers with DVinactive and challenged with DVactive with different serotypes); UnPrDVactive (unprimed mothers with DVinactive, but challenged with DVactive for each serotype, respectively) and Ctrl (mothers that have never been exposed with DV). P-values represent the statistical significance based on *Tukey's Multiple Comparison Test* (*p<0.01;**p< 0.001; ***p<0.0001; ****p< 0.00001; ns: not significant value). Values are expressed as the mean and SE+.

For the number of eggs per individual that was obtained for each treatment, we observed that the control group (offspring of untreated mosquitoes) and the group whose mothers had encountered the active virus (UnPr-DV2active or UnPr-DV4active) had a greater number of eggs per individual on average, whereas individuals of the homologous and heterologous priming groups laid a smaller number of eggs, which was statistically significant in relation to the control and the group that had a single encounter with active virus (Tukey´s multiple comparison test, Ctrl vs. PrHmDV2, p<0.01; Ctrl vs. PrHtDV2, p<0.001; Ctrl vs. PrHmDV4 and PrmHtDV2, p<0.0001) (see **Figure 4A**). It is worth mentioning that the group with heterologous priming (PrHtDV4) had the lower egg-laying rate average (**Figure 4A**). However, the percentage of emergence of priming groups was not significantly different concerning the control or the single encounter with the active virus group (**Figure 4B**).

### The effect of transgenerational immune priming on the percentage of pupation and emergence of adults

**Figure 5:**
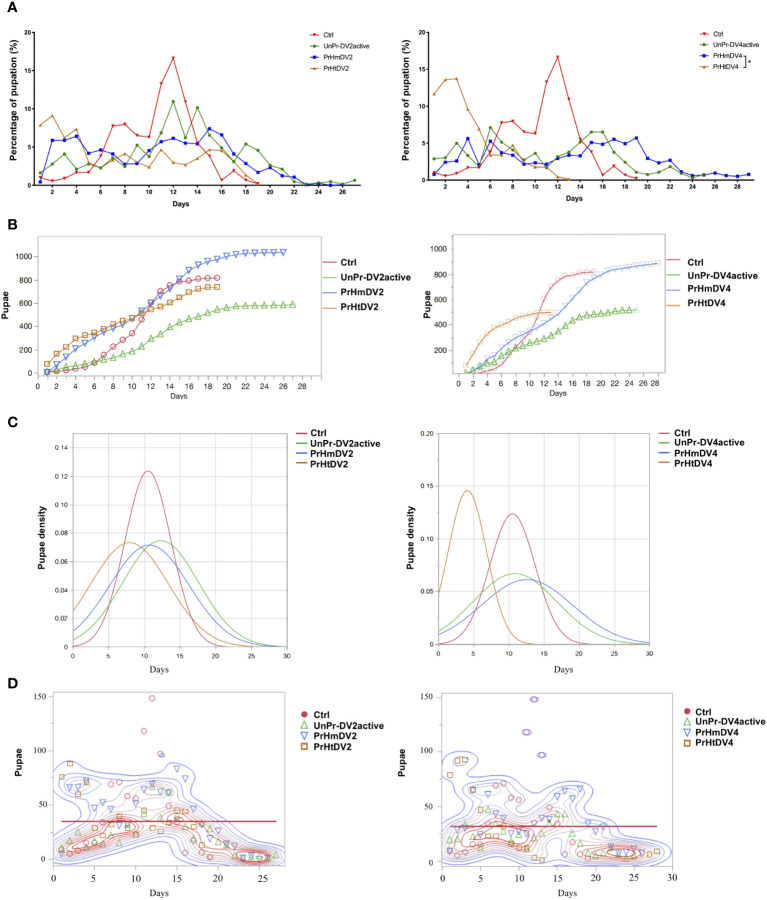
Analysis of the percentage of pupation of progeny (F1) of priming-mothers of Ae. aegyptiper treatment as a function of time. **(A)** Percentage of pupation for each day, **(B)** Number of pupaeaccumulated over time; **(C)** Pupae density over time; **(D)** Pupation events over time. PrHm (priming-mothers with DVinactive and challenged with DVactiv with the same serotype); PrHt (priming-mothers with DVinactive and challenged with DVactive with different serotypes); UnPr-DVactive (unprimed mothers with DVinactive, but challenged with DVactive for each serotype, respectively) and Ctrl (mothers that have never been exposed with DV). *P*-values represent the statistical significancebased on *Tukey's Multiple Comparison* in **(A)** (^*^ *p* < 0.01 ) Chi-square *X^2^* and *Wilcoxon tests* in **(C)** (p < 0.001) **(D)** Mean 34.92, *p*=0.0014^*^ for DV2; mean 31.98, *p*=0.0069^*^ for DV4.

The development of the larvae for each of the mosquitoes from all of the groups was followed from L-1 to L-4, and the time they advanced from the L-4 stage to the pupal stage was regarded as a pupation event and graphed as the percentage of pupation along with the number and density of pupae with respect to time (percentage of larvae that pupated, **Figures 5A–C**). The control and heterologous priming groups finished pupating before day 19 or days before (priming heterologous DV4), whereas the group whose mothers had a single encounter with active DV and the homologous priming group changed from the L-4 phase to the pupal stage in their entirety between days 25 and 29 of development (PrHtDV4 vs. PrHmDV4, chi-squared and Wilcoxon test, p<0.01). Interestingly, the groups with the highest number of pupae corresponded to both homologous groups (800–1,000 pupae per group, **Figure 5B**), whereas the group with a single encounter with active DV had the lowest number of pupae (log-rank between groups for DV2 *X^2 = ^* 10.70, three degrees of freedom, p=0.0134; log-rank between groups for DV4 *X* ^2 = ^16.34, three degrees of freedom, p=0.0010). The density of the pupae (**Figure 5C**) showed differences in the homogeneity of the group; log-rank and Wilcoxon tests demonstrated a statistically significant difference in survival (for DV2, log-rank between groups *X^2 = ^* 243.47, three degrees of freedom, p<0.0001; survival proportion [Peto] *X^2 = ^* 250.0029, three degrees of freedom, p<0.0001; Wilcoxon, p<0.0001). For DV4, the log-rank between groups was *X^2 = ^* 1486.02 (three degrees of freedom, p<0.0001*) and the survival proportion (Peto) was *X^2 = ^* 1313.835 (three degrees of freedom, p<0.0001; *Wilcoxon*, p<0.0001) (statistical comparison between each group, **Supplementary Table 7**). The mean for all groups was between days 11–12, and the average number of pupae per day was 31–34 (**Figure 5D**). However, the density of pupae of the control group and priming heterologous DV4 group reached their peak between days 4 and 11, whereas the homologous group and a single encounter with the active DV group were more homogeneous in terms of the number of pupae over time.

**Figure 6:**
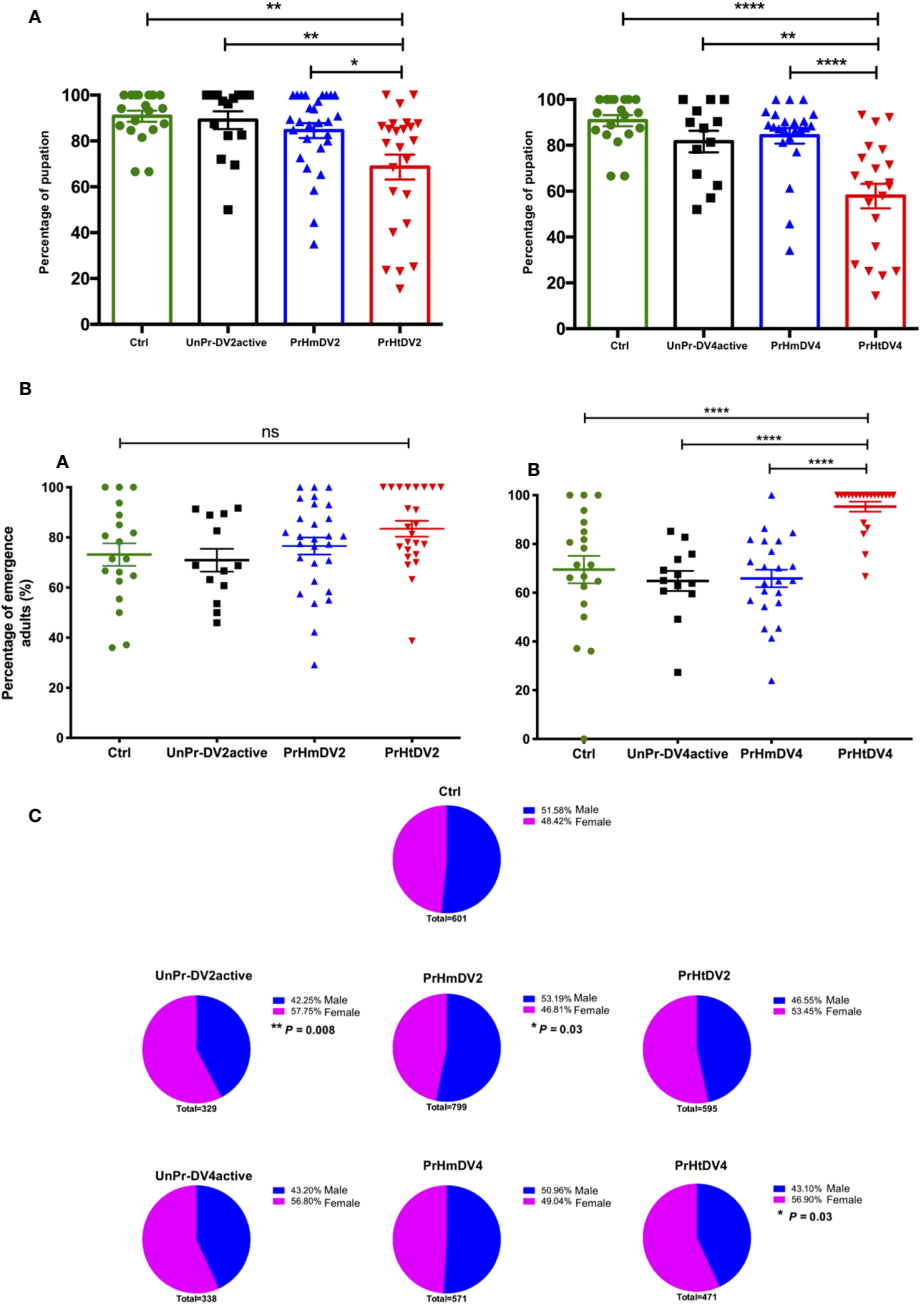
Analysis of **(A)** Percentage of pupation; **(B)** Adult hatching percentage (percentage of pupae that emerge into an adult mosquito) and **(C)** Sex ratio (Males represented in blue vs. Female represented in pink) of progeny (F1) of priming-mothers of Ae. aegypti, previously immunized with dengue virus serotypes 2 (DV2) and 4 (DV4). PrHm (priming-mothers with DVinactive and challenged with DVactive with the same serotype); PrHt (priming-mothers with DVinactive and challenged with DVactive with different serotypes); UnPr-DVactive (unprimed mothers with DVinactive, but challenged with DVactive for each serotype, respectively) and Ctrl (mothers that have never been exposed with DV). P-values of the percentage of pupation and adults hatching represent the statistical significance based on *Tukey's Multiple Comparison Test* (*p<0.01;**p< 0.001; ****p< 0.00001; ns: not significant value). Values are expressed as the mean and SEt. P-values of sex ratio represent the statistical significance based on the Chi-square X2 test.

The number of pupae that reached this stage was obtained for each treatment and divided by the number of eggs of each mosquito that integrated into the respective group, resulting in the percentage of eggs that became pupae. From this, it was observed that the lowest number of positive events (pupation) corresponded to treatments with heterologous priming that showed a reduction of 30–40% in success, indicating a fitness cost (or trade-off) at this stage (Tukey´s multiple comparison test, Ctrl vs. PrHtDV2, p<0.001; Ctrl vs. PrHtDV4, p<0.00001; **Figure 6A**).

Interestingly, and contrary to the percentage of pupation in which the group with the highest percentage corresponded to the heterologous groups, the greatest success in the emergence of adults corresponded to the group whose mothers obtained heterologous priming, which was statistically significant in relation to heterologous group DV4 (Tukey´s multiple comparison test, PrHtDV4 vs. Ctrl, UnPr-DV4active, and PrHmDV4, p<0.00001; **Figure 6B**). The sex ratio of the adult generation (F1) was close to 50%, trending toward a higher percentage of males in the group with homologous priming (PrHmDV2, *X^2^* p=0.03). Interestingly, this trend toward a higher percentage of males was reversed with a clear trend toward a higher percentage of females in groups whose mothers had a single infection with the active virus and the group belonging to mothers with heterologous priming, with a statistically significant difference between those corresponding to immune challenges with DV2 and priming heterologous DV4 (UnPr-DV2active *X^2^*, p= 0.008; PrHtDV2 *X^2^*, p= 0.03, **Figure 6C**). As for the wing measurements from 20 individuals from each group, we found no difference in body size except for the homologous group DV4, which exhibited a slight increase in size (Tukey´s multiple comparison test, p< 0.001, **Figure S4**).

### Effect of transgenerational immune priming on dengue virus susceptibility and the siRNA pathway in the F1 generation

**Figure 7:**
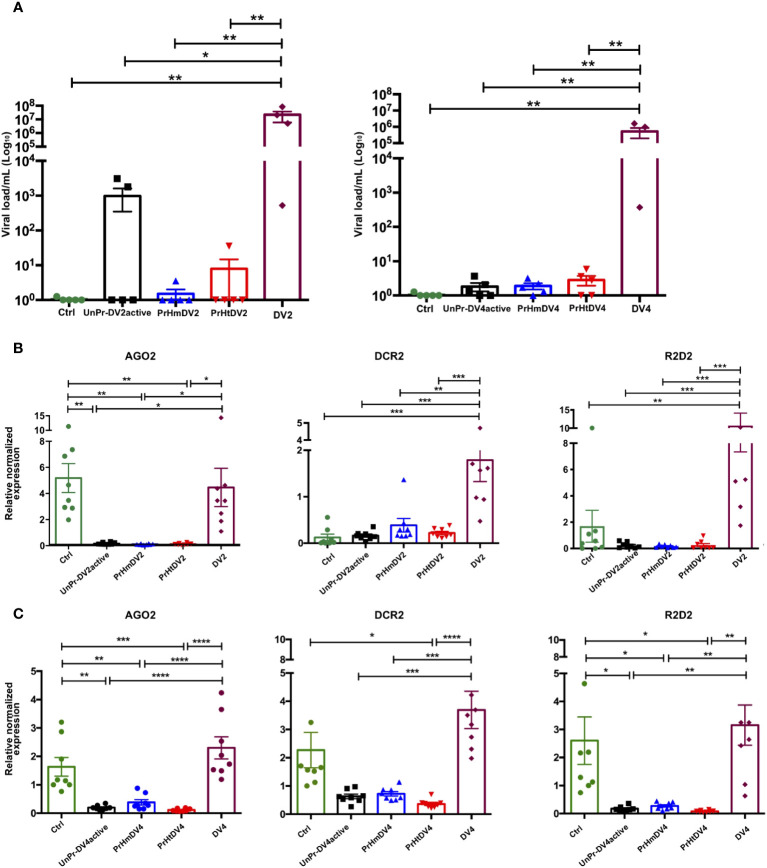
Analysis of **(A)** Viral load of DV and **(B, C)** Expression of relative antiviral immune response genes (siRNA) in progeny (F1) per treatment, at seven days post-infection (dpi) with DV serotype 2 (DV2) and 4 (DV4) respectively. PrHm (priming-mothers with DVinactive and challenged with DVactive with the same serotype); PrHt (priming-mothers with DVinactive and challenged with DVactive with different serotypes); UnPr-DVactive (unprimed mothers with DVinactive, but challenged with DVactive for each serotype, respectively); DV2 (infection control with dengue virus serotype 2); DV4 (infection control with dengue virus serotype 4) and Ctrl (mothers that have never been exposed with DV). P-values represent the statistical significance based on *Tukey's Multiple Comparison Test* (*p<0.01;**p<0.001; ***p<0.0001; ****p< 0.00001). Values are expressed as the mean and SEt. P-values of viral load represent the statistical significance based on Mann-Whitney test.

Once adult mosquitoes were obtained from mothers that were homologously or heterologously immunologically challenged or subjected to a single challenge with active virus, they were exposed again to dengue virus serotype 2 or 4, and the viral load was evaluated subsequently at days 1, 5 (**Figure S5**), and 7 for DV2 and at day 7 for DV4 (**Figure 7**). The viral load of the control positive group DV2, DV4 (mosquitoes fed with blood + DV, whose mothers had never been exposed to viruses) was always higher than the group whose mothers had been exposed to the active DV or those who had homologous priming or UnPr-DV2active (days 1 and 5, Tukey´s multiple comparison test, p< 0.01, **Figure S5**). At 7 days, the trend toward a decrease in viral load in groups whose mothers were exposed to the virus was more evident, with a lower viral load in all groups exposed to DV4 (Un-PrDV4active, PrHmDV4, and PrHtDV4 and statistically significant from the control positive group DV4, Tukey´s multiple comparison test, p<0.001). Additionally, in groups of mosquitoes whose mothers were exposed to immune priming or a single challenge with the active DV (UnPr-DV2active), we found a significant decrease compared with the positive control (Tukey´s multiple comparison test, p< 0.01), although the decrease in viral load was more evident in the group exposed to a single challenge of DV4 (UnPr-DV4active; Tukey´s multiple comparison test, p< 0.001, **Figure 7A**).

The effect of transgenerational immune priming through immune response molecules was evaluated through the relative expression of AGO2, DCR2, and R2D2. Interestingly, the expression of these transcripts was only inducible in groups exposed to the DV2 and DV4 viruses, whose mothers did not have any prior encounters with viruses. Conversely, groups whose mothers experienced homologous or heterologous priming, or exposure to DVactive, had limited siRNA pathway expression 7 days post-infection (**Figures 7B, C**). A second experiment was carried out to examine the viral load and antiviral immune response in mosquitoes following DV infection 5 days post-infection. This trial involved mosquitoes whose mothers had previously encountered DV (Un-PrDV2 active or UnPrDV4active, and PrHm or PrHt DV2 and DV4). The results revealed a similar decrease in viral load in mosquitoes whose mothers had been exposed to DV, as observed 7 days post-infection. Furthermore, the relative expression of genes involved in immune response was similar in both trials (**Figure S6**) with inducible expression by DV2 or DV4 challenge and the inhibition of expression in groups with priming.

The principal components analysis (PCA) did not yield more than 50% of the variance and was not characterized by any of the variables. On the other hand, a correlation analysis for each of the treatments (searching for the relationship between the immune response or viral load with the biological factors evaluated), showed a negative correlation between the viral load and emergence in UnPr-DV2active mosquitoes (r = -0.9416, p=0.0168) and between the viral load and oviposition in PrHtDV2 (r = -0.8877, p=0.044). Likewise, we found a negative correlation between siRNA molecules versus oviposition in UnPr-DV4active mosquitoes (r = -0.7573, p= 0.029), and PrHmDV4 mosquitoes with siRNA molecules versus oviposition and hatching (r = -0.7224, p=0.043; r = -0.8965, p=0.0026. **Figure S7**).

## Discussion

For a long time, it was thought that invertebrates did not have the immunological ability to respond efficiently to a second encounter with a pathogen, in the form of an immune “memory”. However, this has changed in recent years in light of numerous studies documenting the immune responses of different insects to subsequent antigen challenges.

Transgenerational immune priming (TGIP) refers to the transfer of the parental immunological experience to its progeny, which can result in the protection of offspring from repeated encounters with pathogens, such as the dengue virus, which persists in places of endemicity over time and generations of mosquitoes. Numerous studies have shown that previous encounters with a pathogen are likely to impact the fitness of the primed organisms (26, 27, 43–46) (reviewed in (9, 47, 48)).

In this study, we evaluated several parameters of the transgenerational effect on resistance [as recommended by Pigeault et al., 2016 (49),], such as virus load, and the analysis of the effects of TGIP on resistance to dengue virus due to parental virus exposure and the outcome of its infection in offspring, as well as the effects of TGIP on immunity through the RNAi pathway (transgenerational effect on immunity).

It has been reported that *Aedes aegypti* experiences a fitness cost in the presence of dengue virus, which results in lower fecundity and affects longevity and survival (50, 51). Daughters of mothers infected with DV2 have a shorter survival time than those of uninfected mothers, but there were no differences in the number of eggs and the sex ratio (38).

In this study, we observed that, under the context of infection without previous immune priming (UnPr-DV2active or UnPr-DV4active), there were no significant differences in the number of eggs, percentage of hatching, pupation, or mosquito size. However, there were differences in the number of pupae and sex ratio, with a higher proportion of females in the infected group or priming compared with the control group without infection. In contrast to previous research, in which decreased oviposition rates (38, 52) and hatching success were reported in the presence of dengue virus (38), we found that exposure to the virus does not impact hatching and emergence. These differences could be due to the number of viral particles from which the infection was induced. It has been considered that virulence can exert an important factor in the transfer of maternal immunity; when virulence is high, there will be repercussions for longevity and survival (49), as previously observed. In our investigation, we employed lower viral dosages (1x10^6^ RNA copies/ml) than previously documented (3x10^8^ RNA copies/ml [35] or 1x10^9.2^ RNA copies/ml [49]), and in the immune priming design, the virus was inactivated by UV to induce immune priming with a viral load of 1x10^2^ RNA copies/ml. Another crucial factor that must be taken into account is the larger population representation of mosquitoes evaluated in this study compared with previous ones.

On the other hand, and more relevant, is that the groups whose mothers had homologous or heterologous priming, when compared with the control group and the group whose mothers had an encounter with active DV, exhibited significant differences in terms of the number of eggs (smaller but with the same hatching success), percentage of pupation, emergence of adults, and sex ratio (**Figure 6**). Interestingly, the induction of heterologous priming had greater repercussions on the evaluated parameters than those observed with immune priming and a challenge with the same serotype. The two serotypes of DV have a genetic difference of approximately 35% (**Figure S1**); therefore, the immune priming may not be immunologically specific. If the immune response is channeled into the heterogeneity of any viral protein (53), there will be a fitness cost in the biological parameters of the offspring and immune response to the pathogen (54, 55).

Regarding the immune response, we observed that in groups whose mothers had immune priming and those who were exposed twice to active DV had a lower viral load (**Figure 7A**; **Figure S6**), which could constitute a trade-off from the number of oviposited eggs, as has been suggested in other infection models in mosquitoes (34, 56, 57). Mothers exposed to active DV at least once in their lifetime limited their viral load to their daughters compared with previously uninfected controls (**Figure 7A**), thus suggesting that subsequent generations of infected mothers will have greater resistance to DV infections, which is consistent with previous studies of larvae challenged with DV (13).

The mechanism from which TGIP arises is not fully understood. However, it has been proposed that hemocytes, specific RNA sequences, epigenetic factors, siRNAs, and vDNA are integrated into the mosquito genome or DNA synthesis could be involved (58–64). In *Aedes aegypti*, TGIP has been assessed through a chikungunya virus challenge (an alphavirus with ssRNA + genome); however, the mechanism has not been described. In *Drosophila*, it has been suggested that this mechanism may be mediated by gene expression related to the chromatin and DNA binding dependent on viral RNA, but not mediated by the RNAi pathway (23). The RNAi pathway has been reported as an important component of the response to DV (65–67). In this study, we evaluated the involvement of this pathway in the TGIP, through the relative expression of Argonaute 2 (AGO2), Dicer 2 (DCR2), and R2D2. We have observed that the components of this pathway are not involved in the activity of the TGIP, as reported by Mondotte et al. (2020) in *Drosophila* (23) and Ashe et al. (2015) in a model using *Caenorhabditis elegans* (68). It should be noted that some of the transcripts in this pathway have constitutive basal expression, which is expressed without any infection, as previously observed (13). Therefore, it is possible that the transcripts were not detected at the time evaluated in the TGIP groups, and further evaluation is needed at other times. However, in the time window evaluated in the TGIP groups, the viral load was decreased in the priming groups, suggesting that the siRNA pathway has a limited participation in viral regulation in the TGIP. However, it is not ruled out that other pathway cofactors, such as Loquacious (Loqs), could intervene in immune regulation through maternal challenges (69).

The dynamic of dengue transmission is multifactorial, and it converges factors associated with the human population, strain, and circulating virulence of the DV, as well as mosquito vector competition. Dengue cases have constantly fluctuated over the years, reaching peaks in certain seasons before gradually decreasing. In fluctuations of increasing cases of DV, the vector organism becomes relevant; therefore, understanding the immune response of their populations becomes essential to the control or this health problem. In this study, we have observed that previous exposure to an active DV in homologous or heterologous priming significantly decreases viral load; therefore, the mosquito immune response plays an important role in the interaction with DV in TGIP. However, the investment in immunity changes dynamically throughout the host life history and depends mainly on three factors: 1) encounters with the virus (frequency and time scale), 2) virulence of the strains; and 3) the capability of the exerted immune response transmitting to subsequent generations [incidence of increased immunity in the long term (70),]. In geographical areas where the presence of the dengue virus is constant and where more than one viral serotype circulates, two of the three aforementioned assumptions are met. The co-circulation and presence of more than one dengue virus serotype in patients and mosquitoes from different regions have been documented (71–73). Likewise, it has been evaluated that field mosquitoes inoculated with more than one serotype from isolates from patients from Asia and North America can be infected with two serotypes at the same time (74); therefore, co-infections in mosquitoes with more than one viral serotype may be more common than expected. Therefore, there is a potentially heterologous exposure to dengue virus, most likely in areas where more than one serotype circulates at the same time. The immune response exerted by mosquito vector populations is a determinant that must be considered in the dynamics of dengue virus transmission with the investment and cost to mosquitoes of repeated encounters with the same virus. One form of investment involves the induction of a primed immune response that protects the host from re-infection. In addition to the immediate protective effect, immune priming can also provide “delayed” protection against dengue virus through life stages and generations, as examined in this study. Consequently, both types of immune priming have the potential to mediate life history variability in host-pathogen interactions, which could have important consequences for disease prevalence and dynamics, as well as for the demographic structure of the host population (75).

## Conclusion

It has been established that TGIP may offer advantages in terms of resistance to various pathogens (47), although this is not always the case (76, 77). Our research indicates that homologous, heterologous, or DV active challenges in mothers can result in a trade-off in the life history of their daughters, manifesting as a reduction in the time of pupation, an increase in the percentage of pupation, or a shift in the sex ratio, with females being more prevalent in the progeny of mothers with heterologous infection. These factors contribute to greater resistance to dengue virus infections in the daughters. However, owing to our experimental design, it is important to deepen and evaluate the number of generations in which resistance to DV infection is maintained to dilucidate the role of this phenomenon in the transmission dynamics of this arbovirus. Tolerance to infection has been documented as a virus-vector adaptation phenomenon (78, 79); breaking this tolerance by means of immune priming induction can be beneficial. However, this pressure could alter the evolution of more virulent strains, contributing to extended periods of replication and transmission by mosquitoes to human populations, although the current information at hand does not allow the assessment of this phenomenon in open field mosquito populations, so that elucidate the specific mechanism that entails this protection -being the candidates the modifications epigenetics, chromatin rearrangement, through the transfer of siRNAs or even a metabolic shift as seen in trained immunity (80)- or finding markers of previous infections in mosquito populations could lead us to categorize populations of mosquitoes highly potent or resistant to viral infections.

From this work, we could conclude that:

• There is a cost of immune priming on the oviposition of mothers with heterologous infections, although the hatching eggs percentage seems to be costless.
• There is a cost of TGIP on the development time of pupae of the progeny of mothers with heterologous infections, in relation to mothers with homologous infections and mothers with simple infections.
• There is a negative effect on the percentage of pupation in the progeny of mothers with heterologous infections, with the females making this difference. However, 80–90% of pupae emerge as adult mosquitoes.
• There is a significant effect on the sex ratio, with females more abundant than males in the UnPrDV2 and PrHtDV4 groups, whereas the PrHmDV2 group is more abundant in males.
• Daughters of mothers with infections homologous to DV2 have a larger body size compared with those subjected to other treatments.
• There is a significant decrease in transcripts of the siRNA pathway in daughters of mothers exposed to an immune challenge with DV.
• The results showed a lower susceptibility to DV in daughters of mothers previously exposed to the virus compared with those that were untreated.

## Data availability statement

The raw data supporting the conclusions of this article will be made available by the authors, without undue reservation.

## Ethics statement

Ethical review and approval was not required for the study on animals in accordance with the local legislation and institutional requirements.

## Author contributions

JC: Conceptualization, Data curation, Formal Analysis, Investigation, Methodology, Supervision, Validation, Writing – original draft, Writing – review & editing. VV: Conceptualization, Data curation, Investigation, Methodology, Validation, Writing – original draft, Writing – review & editing. JH: Investigation, Methodology, Writing – original draft. EQ: Data curation, Investigation, Methodology, Validation, Writing – review & editing. GD: Methodology, Validation, Writing – original draft. HL: Conceptualization, Supervision, Validation, Visualization, Writing – review & editing.
