# Supplementary material for: The costs of transgenerational immune priming for homologous and heterologous infections with different serotypes of dengue virus in Aedes aegypti mosquitoes
Source: Front Immunol. 2023 Dec 18;14:1286831. doi: 10.3389/fimmu.2023.1286831 (PMC10760805; doi:10.3389/fimmu.2023.1286831)
Supplement: Supplementary file 1 [file DataSheet_1.pdf]

Table 1: Different tests for normal distribution analysis for relative expression of mRNA of antiviral immune response.

Normality of Residuals

		**Test name**	**Statistics**	**P value**	**Passed normality test**
**DV2**	**AGO2**	Anderson-Darling	2.189	<0.0001	No
		D'Agostino-Pearson omnibus	29.28	<0.0001	No
		Shapiro-Wilk	0.8135	<0.0001	No
		Kolmogorov-Smirnov	0.2521	<0.0001	No
	**DCR2**	Anderson-Darling	1.627	0.0003	No
		D'Agostino-Pearson omnibus	38.14	<0.0001	No
		Shapiro-Wilk	0.8053	<0.0001	No
		Kolmogorov-Smirnov	0.1805	0.0021	No
	**R2D2**	Anderson-Darling	1.852	<0.0001	No
		D'Agostino-Pearson omnibus	17.30	0.0002	No
		Shapiro-Wilk	0.8544	0.0001	No
		Kolmogorov-Smirnov	0.2083	0.0001	No
**DV4**	**AGO2**	Anderson-Darling	1.136	0.0050	No
		D'Agostino-Pearson omnibus	11.63	0.0030	No
		Shapiro-Wilk	0.9110	0.0041	No
		Kolmogorov-Smirnov	0.1382	0.0524	No
	**DCR2**	Anderson-Darling	2.236	<0.0001	No
		D'Agostino-Pearson omnibus	29.50	<0.0001	No
		Shapiro-Wilk	0.7970	<0.0001	No
		Kolmogorov-Smirnov	0.2163	<0.0001	No
	**R2D2**	Anderson-Darling	2.240	<0.0001	No
		D'Agostino-Pearson omnibus	17.39	0.0002	No
		Shapiro-Wilk	0.8552	0.0001	No
		Kolmogorov-Smirnov	0.2504	<0.0001	No

Table 2: Different tests for normal distribution analysis for biological parameters for DV2 and DV4.

		Normality of Residuals			
		**Test name**	**Statistics**	**P value**	**Passed normality test**
**DV2**	**Oviposition**	Anderson-Darling	0.2564	0.7162	Yes
		D'Agostino-Pearson omnibus	0.4817	0.7860	Yes
		Shapiro-Wilk	0.9879	0.5509	Yes
		Kolmogorov-Smirnov	0.05243	0.1000	Yes
	**Hatching percentage**	Anderson-Darling	2.578	<0.0001	No
		D'Agostino-Pearson omnibus	9.283	0.0096	No
		Shapiro-Wilk	0.9147	<0.0001	No
		Kolmogorov-Smirnov	0.1284	0.0007	No
	**Percentage of pupation**	Anderson-Darling	3.244	<0.0001	No
		D'Agostino-Pearson omnibus	17.22	0.0002	No
		Shapiro-Wilk	0.8943	<0.0001	No
		Kolmogorov-Smirnov	0.1549	<0.0001	No
	**Percentage of emergence**	Anderson-Darling	0.7865	0.0397	No
		D'Agostino-Pearson omnibus	4.591	0.1007	Yes
		Shapiro-Wilk	0.9599	0.0103	No
		Kolmogorov-Smirnov	0.1060	0.0208	No
**DV4**	**Oviposition**	Anderson-Darling	0.2124	0.8500	Yes
		D'Agostino-Pearson omnibus	0.3104	0.8563	Yes
		Shapiro-Wilk	0.9908	0.8455	Yes

	Kolmogorov-Smirnov	0.06182	0.1000	Yes
**Hatching percentage**	Anderson-Darling	1.608	0.0004	No
	D'Agostino-Pearson omnibus	7.008	0.0301	No
	Shapiro-Wilk	0.9395	0.0009	No
	Kolmogorov-Smirnov	0.1261	0.0031	No
**Percentage of pupation**	Anderson-Darling	2.473	<0.0001	No
	D'Agostino-Pearson omnibus	9.004	0.0111	No
	Shapiro-Wilk	0.9262	0.0003	No
	Kolmogorov-Smirnov	0.1705	<0.0001	No
**Percentage of emergence**	Anderson-Darling	1.707	0.0002	No
	D'Agostino-Pearson omnibus	21.03	<0.0001	No
	Shapiro-Wilk	0.9287	0.0003	No
	Kolmogorov-Smirnov	0.1313	0.0022	No

Table 3: One-way ANOVA analysis and Tukey's multiple comparisons test for biological parameters per treatments (DV2 and DV4).

		ANOVA	SS	DF	MS	F (DFn, DFd)	P value
**DV2**	**Oviposition**						
		Residual	39044	89	438.7		
		Treatment	7679	3	2560	F (3, 89) = 5.834	P=0.0011
	**Hatching percentage**						
		Residual	70418	89	791.2		
		Treatment	2137	3	712.4	F (3, 89) = 0.9003	P=0.4444
	**Percentage of pupation**						
		Residual	6683	3	2228	F (3, 82) = 6.241	P=0.0007
		Treatment	29268	82	356.9		
	**Percentage of emergence**						
		Residual	24505	80	306.3		
		Treatment	1773	3	591.1	F (3, 80) = 1.930	P=0.1314
**DV4**	**Oviposition**						
		Residual	31476	76	414.2		
		Treatment	13898	3	4633	F (3, 76) = 11.19	P<0.0001
	**Hatching**						
		Residual	54213	76	713.3		
		Treatment	925.2	3	308.4	F (3, 76) = 0.4323	P=0.7305
	**Percentage of pupation**						
		Residual	23069	72	320.4		
		Treatment	12642	3	4214	F (3, 72) = 13.15	P<0.0001
	**Percentage of emergence**						
		Residual	22993	73	315.0		
		Treatment	12539	3	4180	F (3, 73) = 13.27	P<0.0001

	Tukey's multiple comparisons test	95.00% CI of diff.	Summary	Adjusted P Value
**Oviposition**	Ctrl vs. UnPr-DV4act	-26.81 to 10.44	ns	0.6573
	Ctrl vs. PrHmDV4	-16.83 to 15.86	ns	0.9998
	Ctrl vs. PrHtDV4	9.864 to 42.55	***	0.0004
	UnPr-DV4act vs. PrHmDV4	-10.42 to 25.82	ns	0.6809
	UnPr-DV4act vs. PrHtDV4	16.27 to 52.52	****	<0.0001
	PrHmDV4 vs. PrHtDV4	10.93 to 42.46	***	0.0002
**Hatching**	Ctrl vs. UnPr-DV4act	-20.66 to 28.24	ns	0.9770
	Ctrl vs. PrHmDV4	-27.29 to 15.61	ns	0.8910
	Ctrl vs. PrHtDV4	-24.72 to 18.18	ns	0.9781
	UnPr-DV4act vs. PrHmDV4	-33.41 to 14.15	ns	0.7126
	UnPr-DV4act vs. PrHtDV4	-30.84 to 16.72	ns	0.8632
	PrHmDV4 vs. PrHtDV4	-18.12 to 23.25	ns	0.9880
**Percentage of pupation**	Ctrl vs. UnPr-DV4act	-7.847 to 26.04	ns	0.4960
	Ctrl vs. PrHmDV4	-8.022 to 21.17	ns	0.6385
	Ctrl vs. PrHtDV4	17.97 to 47.78	****	<0.0001
	UnPr-DV4act vs. PrHmDV4	-18.86 to 13.81	ns	0.9771
	UnPr-DV4act vs. PrHtDV4	7.160 to 40.39	**	0.0019
	PrHmDV4 vs. PrHtDV4	12.09 to 40.51	****	<0.0001
**Percentage of emergence**	Ctrl vs. UnPr-DV4act	-11.95 to 21.29	ns	0.8812
	Ctrl vs. PrHmDV4	-10.60 to 17.93	ns	0.9061
	Ctrl vs. PrHtDV4	-40.39 to -11.24	****	<0.0001
	UnPr-DV4act vs. PrHmDV4	-17.20 to 15.19	ns	0.9984
	UnPr-DV4act vs. PrHtDV4	-46.95 to -14.02	****	<0.0001
	PrHmDV4 vs. PrHtDV4	-43.56 to -15.40	****	<0.0001

**Oviposition**	Ctrl vs. UnPr-DV2act	-16.73 to 20.73	ns	0.9923
	Ctrl vs. PrHmDV2	0.08040 to 32.19	*	0.0484
	Ctrl vs. PrHtDV2	5.869 to 37.53	**	0.0030
	UnPr-DV2act vs. PrHmDV2	-3.411 to 31.68	ns	0.1581
	UnPr-DV2act vs. PrHtDV2	2.358 to 37.04	*	0.0194
	PrHmDV2 vs. PrHtDV2	-8.846 to 19.97	ns	0.7434
**Hatching**	Ctrl vs. UnPr-DV2act	-23.67 to 26.64	ns	0.9987
	Ctrl vs. PrHmDV2	-32.24 to 10.89	ns	0.5676
	Ctrl vs. PrHtDV2	-23.15 to 19.37	ns	0.9955
	UnPr-DV2act vs. PrHmDV2	-35.73 to 11.40	ns	0.5330
	UnPr-DV2act vs. PrHtDV2	-26.67 to 19.91	ns	0.9812
	PrHmDV2 vs. PrHtDV2	-10.57 to 28.14	ns	0.6356
**Percentage of pupation**	Ctrl vs. UnPr-DV2act	-15.43 to 18.79	ns	0.9940
	Ctrl vs. PrHmDV2	-8.538 to 20.91	ns	0.6893
	Ctrl vs. PrHtDV2	6.921 to 37.35	**	0.0015
	UnPr-DV2act vs. PrHmDV2	-11.34 to 20.36	ns	0.8781
	UnPr-DV2act vs. PrHtDV2	4.150 to 36.76	**	0.0079
	PrHmDV2 vs. PrHtDV2	2.165 to 29.73	*	0.0167
**Percentage of emergence**	Ctrl vs. UnPr-DV2act	-14.29 to 18.77	ns	0.9844
	Ctrl vs. PrHmDV2	-17.06 to 10.24	ns	0.9131
	Ctrl vs. PrHtDV2	-24.42 to 3.782	ns	0.2279
	UnPr-DV2act vs. PrHmDV2	-21.07 to 9.756	ns	0.7707
	UnPr-DV2act vs. PrHtDV2	-28.38 to 3.251	ns	0.1670
	PrHmDV2 vs. PrHtDV2	-19.68 to 5.868	ns	0.4916

Table 4: Chi-square test analysis for sex ratio pairwise comparisons for DV2 and DV4.

**Chi-square test**<br>**Sex ratio**<br>**Pairwise comparisons**	**Ctrl vs. UnPr-DV2act**	**Ctrl vs. PrHmDV2**	**Ctrl vs. PrHtDV2**	**Ctrl vs. UnPr-DV4act**	**Ctrl vs. PrHmDV4**	**Ctrl vs. PrHtDV4**
**Chi-square**	6.972	4.294	2.103	3.067	2.070	4.451
**DF**	1	1	1	1	1	1
**P value (two-tailed)**	0.0083	0.0382	0.1470	0.0799	0.1502	0.0349
**P value summary**	**	*	ns	ns	ns	*

Table 5: Two-way ANOVA analysis and Tukey's multiple comparisons test for relative expression mRNA for antiviral immune response per treatment (DV2 and DV4).

DV2	AGO2	ANOVA	SS	DF	MS	F (DFn, DFd)	P value
		Individual	2329	7	332.8	F (7, 28) = 0.5581	P=0.7830
		Treatment	21341	4	5335	F (4, 28) = 8.948	P<0.0001
	DCR2						
		Individual	243.2	7	34.75	F (7, 28) = 0.8748	P=0.8748
		Treatment	1599	4	399.8	F (4, 28) = 10.07	P<0.0001
	R2D2						
		Individual	23374	7	3339	F (7, 28) = 1.363	P=0.2592
		Treatment	77130	4	19283	F (4, 28) = 7.874	P=0.0002
DV4	AGO2						
		Individual	3.258	7	0.4654	F (7, 28) = 1.096	P=0.3925
		Treatment	30.99	4	7.748	F (4, 28) = 18.25	P<0.0001
	DCR2						
		Individual	5.342	7	0.7632	F (7, 28) = 0.5085	P=0.8202
		Treatment	64.41	4	16.10	F (4, 28) = 10.73	P<0.0001
	R2D2						
		Individual	4.872	7	0.6960	F (7, 28) = 0.3045	P=0.9459
		Treatment	71.52	4	17.88	F (4, 28) = 7.822	P=0.0002

	Tukey's multiple comparisons test	95.00% CI of diff.	Summary	Adjusted P Value
**AGO2**	Ctrl vs. UnPr-DV2active	14.61 to 85.75	**	0.0027
	Ctrl vs. PrHmDV2	15.18 to 86.32	**	0.0024
	Ctrl vs. PrHtDV2	15.11 to 86.25	**	0.0024
	Ctrl vs. DV2	-28.33 to 42.81	ns	0.9750
	UnPr-DV2active vs. PrHmDV2	-35.00 to 36.14	ns	>0.9999
	UnPr-DV2active vs. PrHtDV2	-35.07 to 36.07	ns	>0.9999
	UnPr-DV2active vs. DV2	-78.51 to -7.366	*	0.0121
	PrHmDV2 vs. PrHtDV2	-35.64 to 35.50	ns	>0.9999
	PrHmDV2 vs. DV2	-79.08 to -7.934	*	0.0108
	PrHtDV2 vs. DV2	-79.01 to -7.867	*	0.0109
**DCR2**	Ctrl vs. UnPr-DV2active	-9.582 to 8.780	ns	>0.9999
	Ctrl vs. PrHmDV2	-11.79 to 6.572	ns	0.9197
	Ctrl vs. PrHtDV2	-10.14 to 8.217	ns	0.9980
	Ctrl vs. DV2	-25.82 to -7.463	***	0.0001
	UnPr-DV2active vs. PrHmDV2	-11.39 to 6.973	ns	0.9547
	UnPr-DV2active vs. PrHtDV2	-9.743 to 8.618	ns	0.9998
	UnPr-DV2active vs. DV2	-25.42 to -7.062	***	0.0002
	PrHmDV2 vs. PrHtDV2	-7.536 to 10.83	ns	0.9844
	PrHmDV2 vs. DV2	-23.22 to -4.854	**	0.0011
	PrHtDV2 vs. DV2	-24.86 to -6.499	***	0.0003
**R2D2**	Ctrl vs. UnPr-DV2active	-57.56 to 86.62	ns	0.9759
	Ctrl vs. PrHmDV2	-56.93 to 87.25	ns	0.9719
	Ctrl vs. PrHtDV2	-57.75 to 86.43	ns	0.9770
	Ctrl vs. DV2	-169.9 to -25.76	**	0.0040

	UnPr-DV2active vs. PrHmDV2	-71.46 to 72.72	ns	>0.9999
	UnPr-DV2active vs. PrHtDV2	-72.28 to 71.90	ns	>0.9999
	UnPr-DV2active vs. DV2	-184.5 to -40.29	***	0.0009
	PrHmDV2 vs. PrHtDV2	-72.91 to 71.27	ns	>0.9999
	PrHmDV2 vs. DV2	-185.1 to -40.92	***	0.0008
	PrHtDV2 vs. DV2	-184.3 to -40.10	***	0.0009
**AGO2**	Ctrl vs. UnPr-DV4active	0.4868 to 2.385	**	0.0012
	Ctrl vs. PrHmDV4	0.3005 to 2.199	**	0.0054
	Ctrl vs. PrHtDV4	0.5687 to 2.467	***	0.0006
	Ctrl vs. DV4	-1.617 to 0.2815	ns	0.2697
	UnPr-DV4active vs. PrHmDV4	-1.136 to 0.7630	ns	0.9782
	UnPr-DV4active vs. PrHtDV4	-0.8673 to 1.031	ns	0.9991
	UnPr-DV4active vs. DV4	-3.053 to -1.155	****	<0.0001
	PrHmDV4 vs. PrHtDV4	-0.6811 to 1.218	ns	0.9212
	PrHmDV4 vs. DV4	-2.867 to -0.9683	****	<0.0001
	PrHtDV4 vs. DV4	-3.135 to -1.237	****	<0.0001
**DCR2**	Ctrl vs. UnPr-DV4active	-0.1448 to 3.424	ns	0.0833
	Ctrl vs. PrHmDV4	-0.2367 to 3.333	ns	0.1131
	Ctrl vs. PrHtDV4	0.1211 to 3.690	*	0.0319
	Ctrl vs. DV4	-3.209 to 0.3607	ns	0.1670
	UnPr-DV4active vs. PrHmDV4	-1.876 to 1.693	ns	0.9999
	UnPr-DV4active vs. PrHtDV4	-1.519 to 2.051	ns	0.9922
	UnPr-DV4active vs. DV4	-4.848 to -1.279	***	0.0002
	PrHmDV4 vs. PrHtDV4	-1.427 to 2.142	ns	0.9764
	PrHmDV4 vs. DV4	-4.756 to -1.187	***	0.0004
	PrHtDV4 vs. DV4	-5.114 to -1.545	****	<0.0001
**R2D2**	Ctrl vs. UnPr-DV4active	0.2251 to 4.630	*	0.0253
	Ctrl vs. PrHmDV4	0.1262 to 4.531	*	0.0343
	Ctrl vs. PrHtDV4	0.3181 to 4.723	*	0.0189

Ctrl vs. DV4	-2.757 to 1.647	ns	0.9466
UnPr-DV4active vs. PrHmDV4	-2.301 to 2.104	ns	>0.9999
UnPr-DV4active vs. PrHtDV4	-2.109 to 2.295	ns	>0.9999
UnPr-DV4active vs. DV4	-5.185 to -0.7801	**	0.0041
PrHmDV4 vs. PrHtDV4	-2.011 to 2.394	ns	0.9990
PrHmDV4 vs. DV4	-5.086 to -0.6812	**	0.0057
PrHtDV4 vs. DV4	-5.278 to -0.8731	**	0.0030

Table 6: Pairwise comparison using Mann Whitney U test for viral load per treatment (DV2 and DV4)

Mann Whitney test Pairwise comparisons	Ctrl vs. UnPr-DV4act	Ctrl vs. PrHmDV4	Ctrl vs. PrHtDV4	Ctrl vs. DV4	UnPr-DV4act vs. PrHmDV4	UnPr-DV4act vs. PrHtDV4	UnPr-DV4act vs. DV4	PrHmDV4 vs. PrHtDV4	PrHmDV4 vs. DV4	PrHtDV4 vs. DV4
**Mann Whitney U**	6	3	6	0	11	10	0	10	0	0
**P value**	0.1667	*0.0476	0.1667	**0.0079	0.8333	0.6508	**0.0079	0.6905	**0.0079	**0.0079
**Sum of ranks**	21 , 34	18 , 37	21 , 34	15 , 40	26 , 29	25 , 30	15 , 40	25 , 30	15 , 40	15 , 40

Mann Whitney test Pairwise comparisons	Ctrl vs. UnPr-DV2act	Ctrl vs. PrHmDV2	Ctrl vs. PrHtDV2	Ctrl vs. DV2	UnPr-DV2act vs. PrHmDV2	UnPr-DV2act vs. PrHtDV2	UnPr-DV2act vs. DV2	PrHmDV2 vs. PrHtDV2	PrHmDV2 vs. DV2	PrHtDV2 vs. DV2
**Mann Whitney U**	2	0	0	0	9	9	2	12	0	0
**P value**	*0.0317	**0.0079	**0.0079	**0.0079	0.4444	0.4444	*0.0317	>0.9999	**0.0079	**0.0079
**Sum of ranks**	17 , 38	15 , 40	15 , 40	15 , 40	31 , 24	31 , 24	17 , 38	27 , 28	15 , 40	15 , 40
