# Supplementary material for: The costs of transgenerational immune priming for homologous and heterologous infections with different serotypes of dengue virus in Aedes aegypti mosquitoes
Source: Front Immunol. 2023 Dec 18;14:1286831. doi: 10.3389/fimmu.2023.1286831 (PMC10760805; doi:10.3389/fimmu.2023.1286831)
Supplement: Supplementary file 2 [file DataSheet_2.pdf]

A

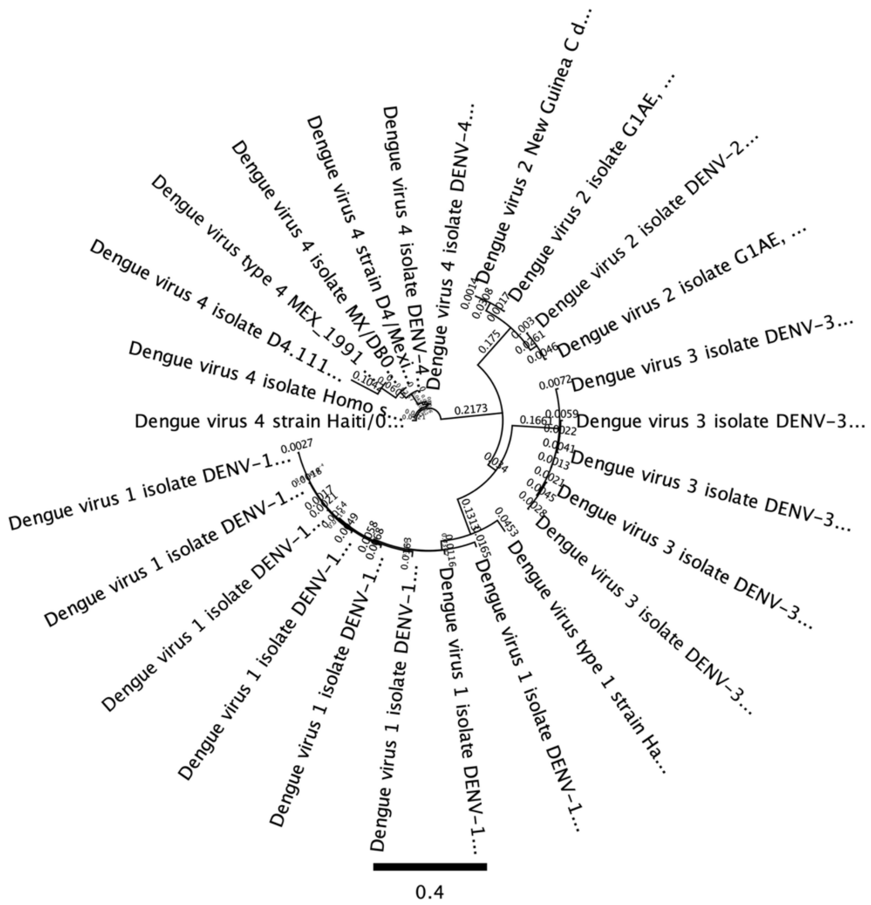

B

	D4_KT27...	D4_MK5...	D4_AY15...	D4_JF804...	D4_HM1...	D4_FJ850...	D4_FJ882...	D2_FJ390...	D2_MH6...	D2_GQ1...	D2_MH7...	D3_EU48...	D3_FJ898...	D3_FJ898...	D3_EU48...	D3_EU48...	D1_DQ6...	D1_EU48...	D1_KJ189...	D1_FJ478...	D1_JQ67...	D1_KJ189...	D1_KJ189...	D1_GU1...	D1_GU1...
D4_KT276273		96.74%	96.70%	96.90%	96.86%	97.43%	97.51%	66.12%	66.14%	66.10%	66.04%	66.08%	65.97%	66.13%	66.14%	66.10%	65.75%	65.76%	65.66%	65.83%	65.67%	65.67%	65.82%	66.01%	65.96%
D4_MK514144	96.74%		97.22%	97.44%	97.34%	97.74%	97.73%	66.13%	66.19%	65.98%	65.95%	66.13%	66.03%	66.15%	66.21%	66.19%	65.66%	65.79%	65.50%	65.79%	65.56%	65.58%	65.72%	65.92%	65.86%
D4_AY152378	96.70%	97.22%		98.38%	97.40%	97.68%	97.68%	66.65%	66.44%	66.49%	66.24%	64.38%	64.23%	64.28%	64.28%	64.02%	64.02%	64.23%	64.69%	64.18%	64.95%	64.74%	64.79%	64.54%	64.69%
D4_JF804055	96.90%	97.44%	98.38%		98.63%	98.45%	98.18%	66.46%	66.26%	66.46%	66.26%	63.50%	63.03%	63.16%	63.10%	63.23%	63.84%	64.78%	64.98%	64.31%	64.98%	64.92%	65.05%	64.92%	64.98%
D4_HM171571	96.86%	97.34%	97.40%	98.63%		98.70%	98.43%	66.46%	66.26%	66.26%	66.05%	63.80%	63.32%	63.46%	63.39%	63.52%	63.66%	64.41%	64.55%	64.07%	64.55%	64.62%	64.75%	64.48%	64.55%
D4_FJ850059	97.43%	97.74%	97.68%	98.45%	98.70%		98.96%	66.17%	66.11%	66.03%	65.99%	66.04%	65.99%	66.10%	66.12%	66.06%	65.57%	65.66%	65.45%	65.68%	65.49%	65.50%	65.66%	65.82%	65.78%
D4_FJ882601	97.51%	97.73%	97.68%	98.18%	98.43%	98.96%		66.14%	66.07%	66.05%	66.02%	66.02%	65.98%	66.12%	66.15%	66.09%	65.58%	65.67%	65.47%	65.69%	65.53%	65.53%	65.69%	65.85%	65.83%
D2_FJ390389.1	66.12%	66.13%	66.65%	66.46%	66.46%	66.17%	66.14%		99.67%	94.40%	93.87%	68.27%	68.15%	68.18%	68.18%	68.17%	68.13%	68.05%	67.42%	67.95%	67.52%	67.48%	67.66%	67.73%	67.77%
D2_MH613985	66.14%	66.19%	66.44%	66.26%	66.26%	66.11%	66.07%	99.67%		94.34%	93.81%	68.26%	68.16%	68.19%	68.21%	68.20%	68.30%	68.09%	67.36%	68.01%	67.45%	67.41%	67.57%	67.77%	67.80%
D2_GQ199893	66.10%	65.98%	66.49%	66.46%	66.26%	66.03%	66.05%	94.40%	94.34%		99.25%	68.37%	68.37%	68.41%	68.33%	68.37%	68.32%	68.39%	67.55%	68.22%	67.68%	67.60%	67.82%	68.01%	67.99%
D2_MH781014	66.04%	65.95%	66.24%	66.26%	66.05%	65.99%	66.02%	93.87%	93.81%	99.25%		68.26%	68.27%	68.31%	68.24%	68.25%	68.29%	68.32%	67.56%	68.15%	67.68%	67.60%	67.80%	67.94%	67.94%
D3_EU482555	66.08%	66.13%	64.38%	63.50%	63.80%	66.04%	66.02%	68.27%	68.26%	68.37%	68.26%		98.17%	98.50%	98.81%	98.39%	72.00%	71.97%	71.59%	71.92%	71.70%	71.52%	71.70%	72.05%	72.05%
D3_FJ898442	65.97%	66.03%	64.23%	63.03%	63.32%	65.99%	65.98%	68.15%	68.16%	68.37%	68.27%	98.17%		99.01%	98.61%	98.18%	71.83%	71.90%	71.45%	71.80%	71.63%	71.42%	71.57%	71.95%	71.91%
D3_FJ898440	66.13%	66.15%	64.28%	63.16%	63.46%	66.10%	66.12%	68.18%	68.19%	68.41%	68.31%	98.50%	99.01%		99.03%	98.60%	71.99%	71.88%	71.60%	71.80%	71.70%	71.49%	71.68%	72.03%	72.02%
D3_EU482559	66.14%	66.21%	64.28%	63.10%	63.39%	66.12%	66.15%	68.18%	68.21%	68.33%	68.24%	98.81%	98.61%	99.03%		99.51%	71.86%	71.86%	71.54%	71.82%	71.59%	71.45%	71.60%	71.96%	71.94%
D3_EU482595	66.10%	66.19%	64.02%	63.23%	63.52%	66.06%	66.09%	68.17%	68.20%	68.37%	68.25%	98.39%	98.18%	98.60%	99.51%		71.89%	71.90%	71.58%	71.81%	71.61%	71.49%	71.64%	71.99%	71.98%
D1_DQ672563	65.75%	65.66%	64.02%	63.84%	63.66%	65.57%	65.58%	68.13%	68.30%	68.32%	68.29%	72.00%	71.83%	71.99%	71.86%	71.89%		91.42%	91.20%	91.96%	91.30%	91.07%	91.34%	91.62%	91.62%
D1_EU482567	65.76%	65.79%	64.23%	64.78%	64.41%	65.66%	65.67%	68.05%	68.09%	68.39%	68.32%	71.97%	71.90%	71.88%	71.86%	71.90%	91.42%		96.03%	97.19%	96.45%	96.40%	96.67%	96.87%	96.90%
D1_KJ189367	65.66%	65.50%	64.69%	64.98%	64.55%	65.45%	65.47%	67.42%	67.36%	67.55%	67.56%	71.59%	71.45%	71.60%	71.54%	71.58%	91.20%	96.03%		96.72%	97.28%	97.22%	97.33%	97.49%	97.49%
D1_FJ478457	65.83%	65.79%	64.18%	64.31%	64.07%	65.68%	65.69%	67.95%	68.01%	68.22%	68.15%	71.92%	71.80%	71.80%	71.82%	71.81%	91.96%	97.19%	96.72%		97.12%	96.99%	97.24%	97.50%	97.52%
D1_JQ675358	65.67%	65.56%	64.95%	64.98%	64.55%	65.49%	65.53%	67.52%	67.45%	67.68%	67.68%	71.70%	71.63%	71.70%	71.59%	71.61%	91.30%	96.45%	97.28%	97.12%		98.46%	98.78%	98.88%	99.06%
D1_KJ189368	65.67%	65.58%	64.74%	64.92%	64.62%	65.50%	65.53%	67.48%	67.41%	67.60%	67.60%	71.52%	71.42%	71.49%	71.45%	71.49%	91.07%	96.40%	97.22%	96.99%	98.46%		99.31%	99.17%	98.89%
D1_KJ189369	65.82%	65.72%	64.79%	65.05%	64.75%	65.66%	65.69%	67.66%	67.57%	67.82%	67.80%	71.70%	71.57%	71.68%	71.60%	71.64%	91.34%	96.67%	97.33%	97.24%	98.78%	99.31%		99.46%	99.18%
D1_GU131956	66.01%	65.92%	64.54%	64.92%	64.48%	65.82%	65.85%	67.73%	67.77%	68.01%	67.94%	72.05%	71.95%	72.03%	71.96%	71.99%	91.62%	96.87%	97.49%	97.50%	98.88%	99.17%	99.46%		99.41%
D1_GU131960	65.96%	65.86%	64.69%	64.98%	64.55%	65.78%	65.83%	67.77%	67.80%	67.99%	67.94%	72.05%	71.91%	72.02%	71.94%	71.98%	91.62%	96.90%	97.49%	97.52%	99.06%	98.89%	99.18%	99.41%	

Supplementary Figure 1. Phylogenetic tree of sequences of the four dengue virus serotypes. **A**) Circular tree (genetic distance model- Tamura-Nei) method Neighbor-joining. We present the genetic relationships between the four dengue virus serotypes using isolated sequences obtained in Mexico or USA. The study incorporates isolates from DV4 patients and the reference strain of DV2 New Guinea (Fj390389.1). Additionally, a matrix of distances **B**) between the four serotypes of dengue virus is displayed, highlighting a genetic distance of 66% between DV2 and DV4.

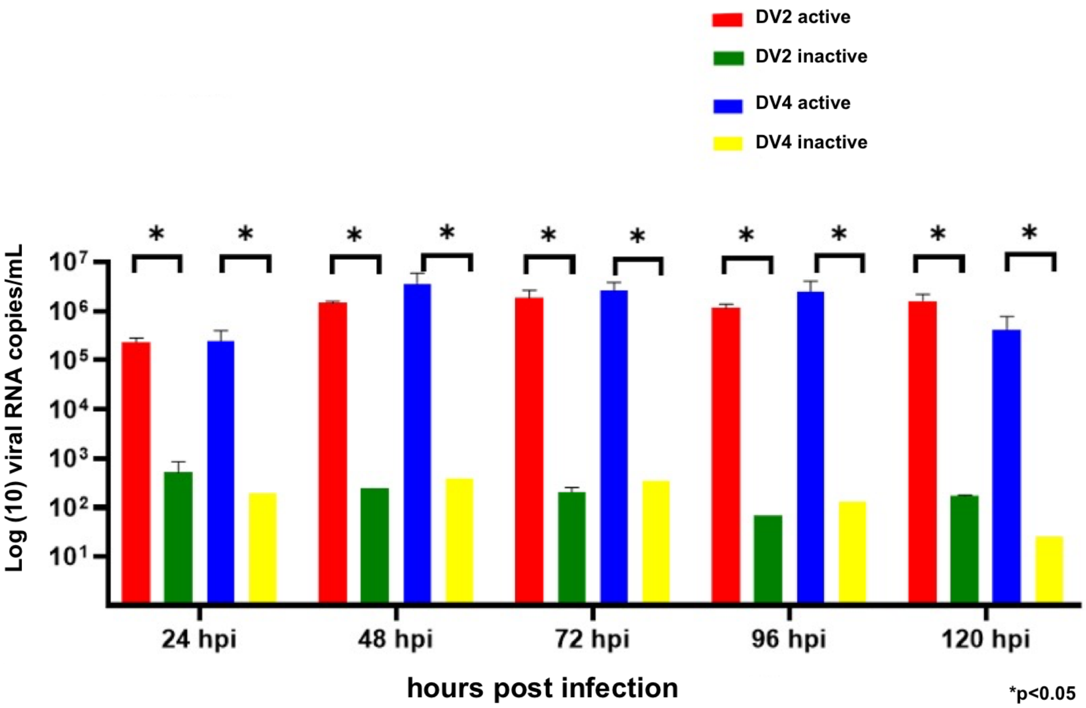

Supplementary Figure 2. Kinetics of C6/36 cell infection with dengue virus serotypes 2 and 4. The graph shows on the Y axis the number of copies of viral RNA/ml. detected in the supernatant of C6/36 cells infected with dengue virus serotypes 2 (DV2) and 4 (DV4), while the *X* axis shows the time points at which each of the supernatants of these infected cells were collected. The first two bars at each time point correspond to infection with DV2 in active state (red color) and in state exposed to UV light for 3 hours (green color). The third bar at each time point (blue) corresponds to the infection made in C6/36 cells infected with DV4 in the active state, while the bar in yellow represents the supernatant of C6/36 cells exposed to DV4 that was previously irradiated with UV light. C6/36 cell infection data were analyzed by applying a *2-way ANOVA of multiple comparisons of Tukey*. The statistical test was subject to a value of *$p<0.05$* for a significant difference. The data were analyzed and plotted in the *GraphPad Prism version 6.1 program*.

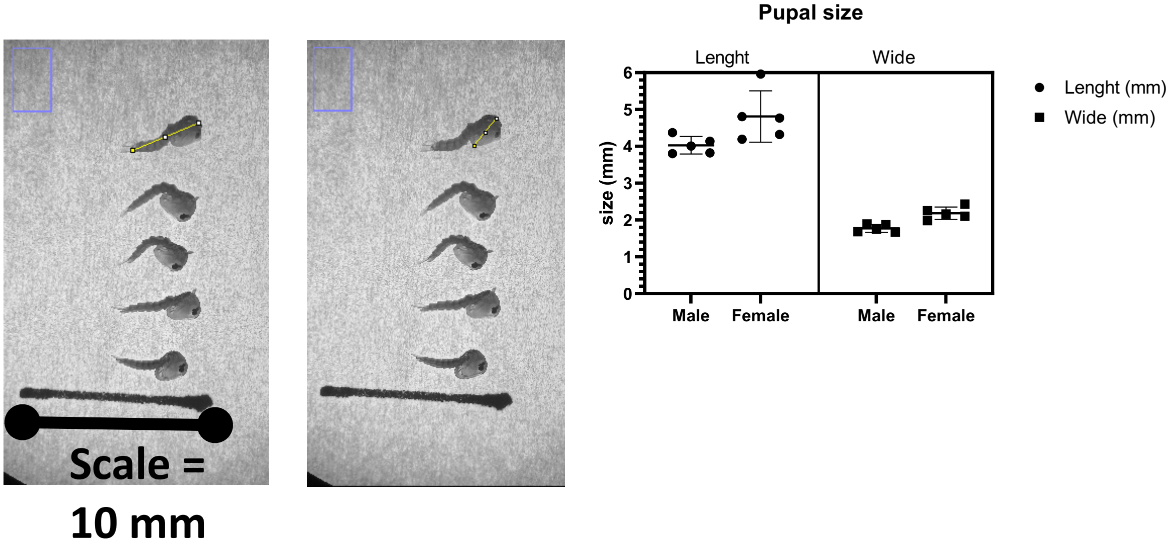

Supplementary Figure 3. Body measurements of 5 pupae per sex. The width and length of the pupae were measured with the help of the ImageJ program, using a 10 mm scale as a reference.

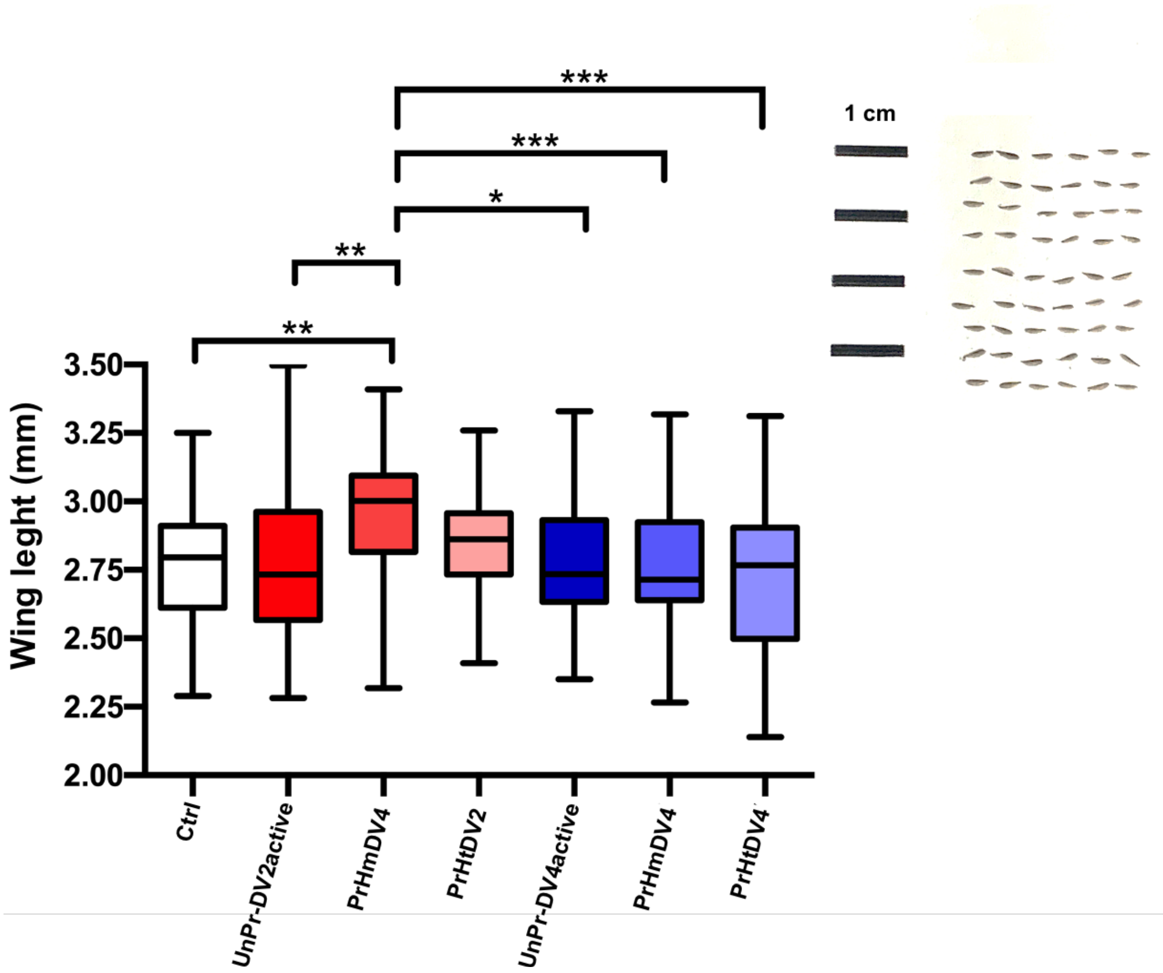

Supplementary Figure 4. Wing length of 30 individuals for each of the treatments, showing significant differences between the UnPrDV2active group and the rest of the groups. *P*-values represent the statistical significance based on *Tukey's Multiple Comparison Test* (**p*< 0.01; ***p*< 0.001).

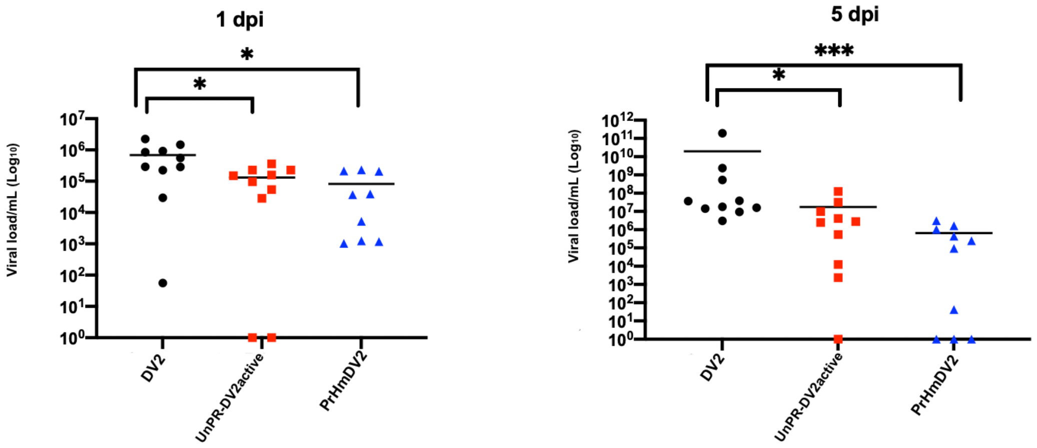

Supplementary Figure 5. Analysis of Viral load of DV in progeny (F1), at one and five days post-infection (dpi) with DV2. *P*-values represent the statistical significance based on *Tukey's Multiple Comparison Test* (*p< 0.01; ***p< 0.0001).

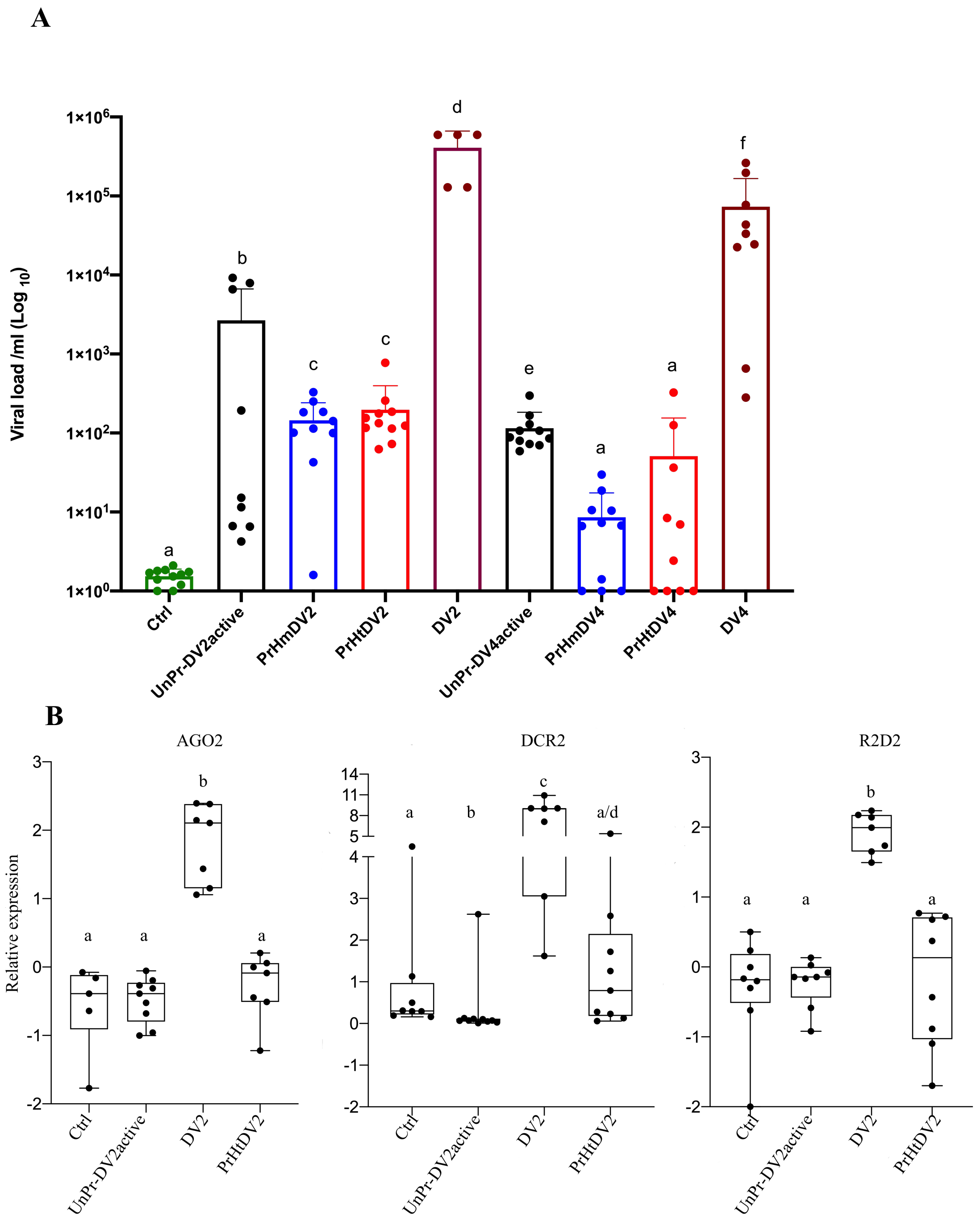

Supplementary Figure 6. Analysis of **A)** Viral load of DV and **B)** Relative expression of antiviral immune response genes [siRNA: Argonaute (AGO2), Dicer 2 (DCR2) and R2D2] in progeny (F1) per treatment, at five days post-infection (dpi) with DV serotype 2 (DV2) and 4 (DV4). PrHm (priming-mothers with DVinactive and challenged with DVactive with the same serotype); PrHt (priming-mothers with DVinactive and challenged with DVactive with different serotypes); UnPr-DVactive (not priming-mothers with DVinactive, but challenged with DVactive for each serotype, respectively); DV2 (infection control with dengue virus serotype 2); DV4 (infection control with dengue virus serotype 4) and Ctrl (mothers that have never been exposed with DV). *P*-values represent the statistical significance based on *Mann-Whitney U test;* different letters represent significant values (*p<0.05*), while equal letters are non-significant values.

A

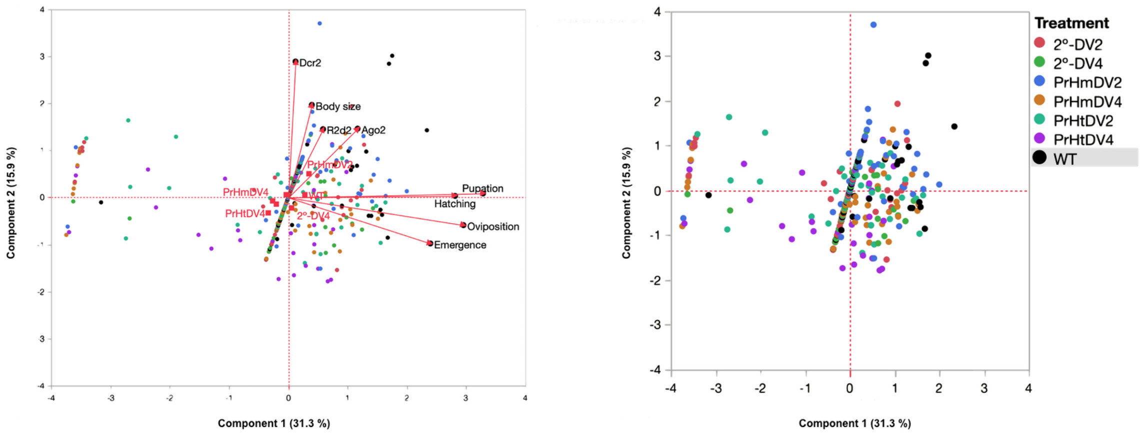

B

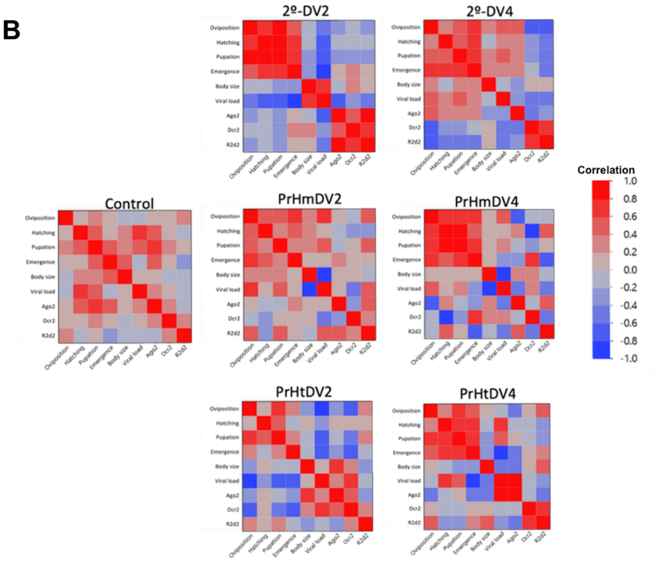

Treatment	Variable	Per variable	r	P < value
2º-DV2	Hatching	Oviposition	0.6923	0.0007*
	Pupation	Oviposition	0.8868	<.0001*
	Pupation	Hatching	0.8582	<.0001*
	Emergence	Oviposition	0.5119	0.0210*
	Emergence	Hatching	0.6239	0.0033*
	Emergence	Pupation	0.6279	0.0030*
	Viral load	Emergence	-0.9416	0.0168*
	R2d2	Ago2	0.9434	0.0004*
	R2d2	Dcr2	0.7699	0.0255*
PrHmDV2	Hatching	Oviposition	0.4343	0.0165*
	Pupation	Oviposition	0.5310	0.0025*
	Emergence	Oviposition	0.6055	0.0004*
	Emergence	Hatching	0.5146	0.0036*
	Body size	Hatching	0.3750	0.0412*
PrHtDV2	Pupation	Oviposition	0.6311	0.0002*
	Pupation	Hatching	0.5897	0.0006*
	Viral load	Oviposition	-0.8877	0.0444*
	Ago2	Body size	0.7438	0.0344*
	Dcr2	Oviposition	-0.7167	0.0455*
Control	Emergence	Pupation	0.4729	0.0352*

Treatment	Variable	Per variable	r	P < value
2º-DV4	Pupation	Oviposition	0.5387	0.0383*
	Emergence	Oviposition	0.6173	0.0142*
	Emergence	Hatching	0.6313	0.0116*
	Emergence	Pupation	0.6721	0.0061*
	R2d2	Oviposition	-0.7573	0.0296*
PrHmDV4	Hatching	Oviposition	0.7455	<.0001*
	Pupation	Oviposition	0.7860	<.0001*
	Pupation	Hatching	0.8261	<.0001*
	Emergence	Oviposition	0.7402	<.0001*
	Emergence	Hatching	0.5604	0.0013*
	Emergence	Pupation	0.7809	<.0001*
	Ago2	Oviposition	-0.7224	0.0430*
	Dcr2	Hatching	-0.8965	0.0026*
PrHtDV4	Pupation	Oviposition	0.6011	0.0012*
	Pupation	Hatching	0.7195	<.0001*
	Emergence	Oviposition	0.5299	0.0054*
	Emergence	Hatching	0.6280	0.0006*
	Emergence	Pupation	0.6854	0.0001*
	Ago2	Viral load	0.9242	0.0248*

Supplementary Figure 7. **A)** Principal Analysis Components (PCA), **B)** Correlation matrix by treatment blue negative correlation, red positive correlation. *P*-values represent the statistical significance based on the *Chi-square* X^2 *test* (**p< 0.01*). In the table, in red, we show the correlation between immune factors and biological factors.
